# Hypertonic stress induced changes of *Pseudomonas fluorescens* adhesion towards soil minerals studied by AFM

**DOI:** 10.1038/s41598-023-44256-7

**Published:** 2023-10-10

**Authors:** Abd Alaziz Abu Quba, Marc-Oliver Goebel, Mariam Karagulyan, Anja Miltner, Matthias Kästner, Jörg Bachmann, Gabriele E. Schaumann, Doerte Diehl

**Affiliations:** 1 Institute for Environmental Sciences, University of Kaiserslautern-Landau (RPTU), Landau, Germany; 2 Institute of Soil Science, Leibniz Universität Hannover, Hannover, Germany; 3 Department of Environmental Biotechnology, Helmholtz Centre for Environmental Research – UFZ, Leipzig, Germany

**Keywords:** Biogeochemistry, Environmental sciences

## Abstract

Studying bacterial adhesion to mineral surfaces is crucial for understanding soil properties. Recent research suggests that minimal coverage of sand particles with cell fragments significantly reduces soil wettability. Using atomic force microscopy (AFM), we investigated the influence of hypertonic stress on *Pseudomonas fluorescens* adhesion to four different minerals in water. These findings were compared with theoretical XDLVO predictions. To make adhesion force measurements comparable for irregularly shaped particles, we normalized adhesion forces by the respective cell-mineral contact area. Our study revealed an inverse relationship between wettability and the surface-organic carbon content of the minerals. This relationship was evident in the increased adhesion of cells to minerals with decreasing wettability. This phenomenon was attributed to hydrophobic interactions, which appeared to be predominant in all cell–mineral interaction scenarios alongside with hydrogen bonding. Moreover, while montmorillonite and goethite exhibited stronger adhesion to stressed cells, presumably due to enhanced hydrophobic interactions, kaolinite showed an unexpected trend of weaker adhesion to stressed cells. Surprisingly, the adhesion of quartz remained independent of cell stress level. Discrepancies between measured cell–mineral interactions and those calculated by XDLVO, assuming an idealized sphere-plane geometry, helped us interpret the chemical heterogeneity arising from differently exposed edges and planes of minerals. Our results suggest that bacteria may have a significant impact on soil wettability under changing moisture condition.

## Introduction

Adhesion to solid surfaces plays a crucial role for bacteria^1^, resulting from a complex interplay of physicochemical interactions including electrostatic, van der Waals and Lewis acid–base (AB) forces^2,3^. In soil ecosystems, bacteria exert a significant influence on various soil properties and soil functions. For instance, their capacity to colonize surfaces contributes to soil aggregate formation and mineral weathering^4^. Even a slight increase in microbial biomass or its residues at mineral surfaces can lead to substantial changes in surface characteristics, such as surface roughness, surface charge, adsorption affinities and wettability^5^. Consequently, bacterial adhesion is of major importance regarding soil functions in soil ecosystems^6,7^.

The majority of soil bacteria regularly experience suboptimal growth conditions due to competition for resources, or changing environmental factors, putting them nearly constantly under stress. With climate change, particularly drought stress will become increasingly important for the soil microbial community^8^. Surprisingly, there are currently no studies available that have analyzed the impact of drought stress on the adhesion of bacterial cells to minerals. Hence, our goal is to investigate the role of bacterial cells and their cell wall remnants in shaping the dynamics of mineral surface properties in soil under changing moisture conditions. In a recent study, we demonstrated that subjecting *Pseudomonas fluorescens* cells to hypertonic stress induced by NaCl reduces their wettability^9^. We now seek to explore whether soil bacteria and their cell envelopes not only decrease the wettability of soil particles after dry periods but also exhibit stronger adhesion to surfaces, making them less susceptible to degradation, ultimately leading to an increased persistence of soil water repellency.

In liquid media, the aggregation or dispersion behavior of cell-mineral associations can be described using the extended Derjaguin–Landau–Verwey–Overbeek (XDLVO) theory^2,4,10^. However, XDLVO calculations necessitate geometrically well-defined interfaces with uniform surface charge density, whereas real colloidal systems in soil display morphological and chemical variations that can lead to significant deviations from theoretical models with simplified geometrical shapes (e.g., spherical and planar approximation)^11–13^. Numerical methods such as surface element integration (SEI) can determine the precise interaction forces between different any shapes and a flat surface^14,15^. However, considering chemical heterogeneity arising from structural edge effects of minerals demands substantial computational power^15^ and, to our knowledge, has not been applied yet. Consequently, it is imperative to directly measure adhesion forces under the most realistic conditions.

Atomic force microscopy (AFM) serves as a powerful tool for investigating cell-mineral interactions (CMI)^4,16^ and is the only technique with nano-resolution applicable to living cells in aqueous solutions^17^. In examining the interaction between *E. coli* and “flat” single crystals of muscovite, goethite and graphite, it was revealed that electrostatic forces predominantly dictate the adhesion forces’ polarity. Simultaneously, both the surface hydrophobicity and roughness of the minerals exert a direct influence on the absolute force magnitude within the attractive force regimes^18^. When dealing with irregularly shaped natural substances, tip-sample interactions become increasingly complex due to the effect of contact area, and the potential for contact at multiple sites. Adhesion measurements involving *E. coli*-coated tipless probes interacting with hematite or corundum nanoparticles have demonstrated that smaller-radius particles adhere more strongly to the cells due to a greater number of tip-sample contact sites compared to larger particles^19^. Consistently, *E. coli* cells displayed stronger adhesion to needle-like goethite particles than to flat goethite surfaces^4^.

Hence, adhesion values without information on the contact area pose interpretation challenges, rendering quantitative comparison of interaction forces between various strains and substrates unfeasable^20–22^. Consequently, the adhesion pressure, defined as the normalized adhesion force over the corresponding tip-sample contact area, was introduced^16^. This parameter facilitates a quantitative comparison of interaction data^16^. A novel protocol for characterizing irregular interface geometries formed by natural mineral particles and cells enables the measurement of CMI in a liquid medium^23^.

In addition to the contact area, the orientation at which a mineral particle contacts the cell surface likely influences adhesion. Some minerals exhibit anisotropic crystallographic structure^24–28^, such as goethite which comprises double chains of Fe-octahedra connected via hydrogen bonds across the long axis of the particle^25^. The edges, despite representing only 2–5% of the particle surface, contain a higher density of potential hydrogen bonding sites per unit area compared to the particle planes^24,25^. Similarly, montmorillonite and kaolinite possess additional polar sites along their edges with octahedral Al–OH and tetrahedral Si–OH groups located at the edges of the particle rather than on the basal planes which are terminated by either one of these groups for kaolinite and by Si–OH for montmorillonite, respectively^26,27^. Larger quartz grains have irregularly shaped surfaces that become hydroxylated in water facilitating hydrogen bonding with lipopolysaccharides of Gram-negative bacteria^28^.

To investigate the effect of growth under drought stress on bacterial cell adhesion to minerals, we tested four hypotheses:

1. Since cells are likely slightly negatively charged at the experimental pH of 5.9, we anticipate jump-to-contact events in force spectroscopy for positively charged minerals indicating an attractive force^4^ and repulsive electrostatic forces when negatively charged mineral tips approach cell surfaces.
2. Mineral wettability influences adhesion pressures towards cells. More wettable minerals adhere to bacteria to a lesser extent as a polar aqueous medium readily wet hydrophilic surfaces during cell-mineral separation.
3. The increased protein content (hydrophobic surface domains) on stressed cell surfaces compared to unstressed cells^9^ makes adhesion to mineral surfaces in a polar aqueous medium more energetically favorable for stressed cells resulting in an increased adhesion pressure.
4. The non-uniform distribution of functional groups and charge on kaolinite surfaces^4^ generally leads to less agreement between AFM spectroscopy results and XDLVO theory for the interaction with stressed and unstressed bacteria compared to quartz, montmorillonite and goethite.

To assess these hypotheses, we conducted direct single cell-mineral interaction studies using AFM. Specifically, we measured force-distance (FD) curves towards *P. fluorescens* cells grown under hypertonic stress or under unstressed conditions using probes modified with kaolinite, montmorillonite, goethite or quartz particles. The minerals used represent a diverse range of particle shapes and physicochemical properties. Our experiments thus encompass the complexity of interactions with the typical Gram-negative soil bacterium *P. fluorescens*. Adhesion forces were normalized to the real 3D contact area. Surface roughness extends the range and depth of the secondary minimum while decreasing the energy barrier’s magnitude^29^. Furthermore, morphological heterogeneity increases attractive interactions in the primary minimum compared to XDLVO calculations using simplified geometrical models^15^. Hence, we only qualitatively compared the measured adhesion pressures with theoretical XDLVO energy profiles. In these models, minerals and cells were approximated as spheres and planes, respectively, using macroscopic parameters like contact angle and zeta potential. Any deviations were attributed to mineral shapes and potential chemical heterogeneity resulting from differently exposed clay edges and planes when interacting with bacterial cells. This approach enabled the discussion of potential relationships between surface charge, crystal structure, wettability, and chemical composition of minerals with the adhesion pressure of specific cell-mineral pairs.

## Results

### Characteristics of the minerals and their aggregates

**Figure 1:**
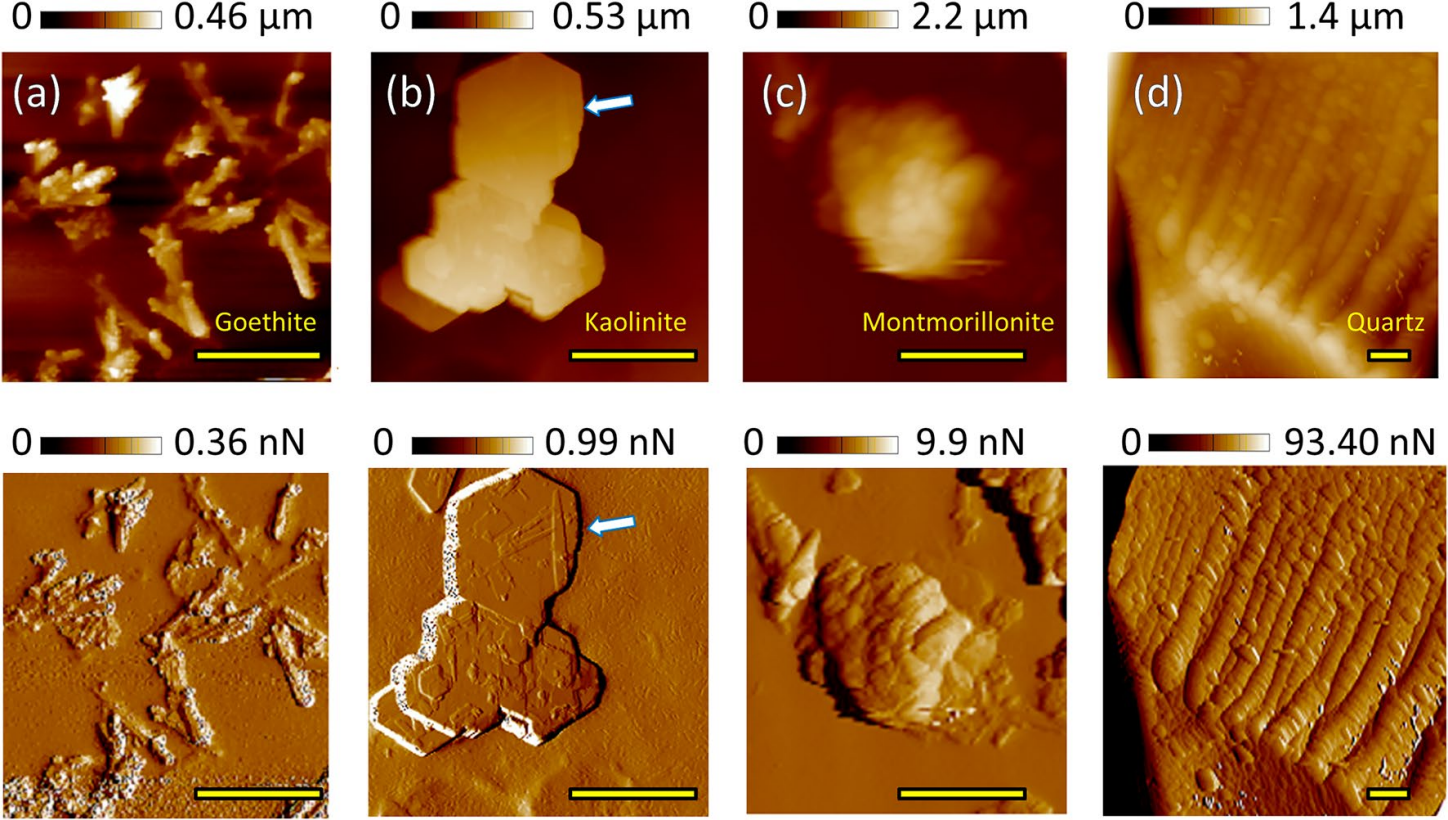
AFM Height (top) and Peak Force Error (bottom) maps of (**a**) goethite, (**b**) kaolinite, (**c**) montmorillonite and (**d**) quartz, obtained in KNO_3_ solution with sharp tips. The scale bar is 1.5 µm for all images.

To provide an overview of the size, shape and aggregation behavior of the minerals used, Fig. 1a–c present Height and Peak Force Error images of goethite, kaolinite and montmorillonite, respectively. Figure 1d displays an image of the edge of a quartz particle. More detailed images can be found in Supplementary Fig. S-I 1. The appearance of goethite and kaolinite notably reflect their crystal structures (Fig. 1a,b). While many kaolinite particles exhibit a flat shape with a nominal average size of ~ 500 nm and aggregate in form of smooth plates with sharp edges, others exhibit the typical pseudo-hexagonal shape^26^ (indicated by arrows in Fig. 1b and Supplementary Fig. S-I 1). Goethite crystals with a nominal size of 100 × 800 nm can be identified by their needle-like shape in individual particles with flat orientations or within aggregated structures where some needles protrude from goethite clusters. Montmorillonite particles averaging around 400 nm in size formed the largest and highest aggregates with an exfoliated morphology (Fig. 1c and Supplementary Fig. S-I 2). Conversely, larger quartz grains exhibit irregular shapes (Fig. 1d). Consequently, the small and relatively smooth goethite particles and the smoothest kaolinite aggregates possess smaller and less variable roughness R_q_ values of 18 ± 9 nm (n = 150) and 11 ± 9 nm (n = 80), respectively. In contrast, montmorillonite aggregates and quartz particles have higher roughness values of 141 ± 88 nm (n = 100) and 119 ± 126 nm (n = 60), respectively.

### Characteristics of the modified tips

**Figure 2:**
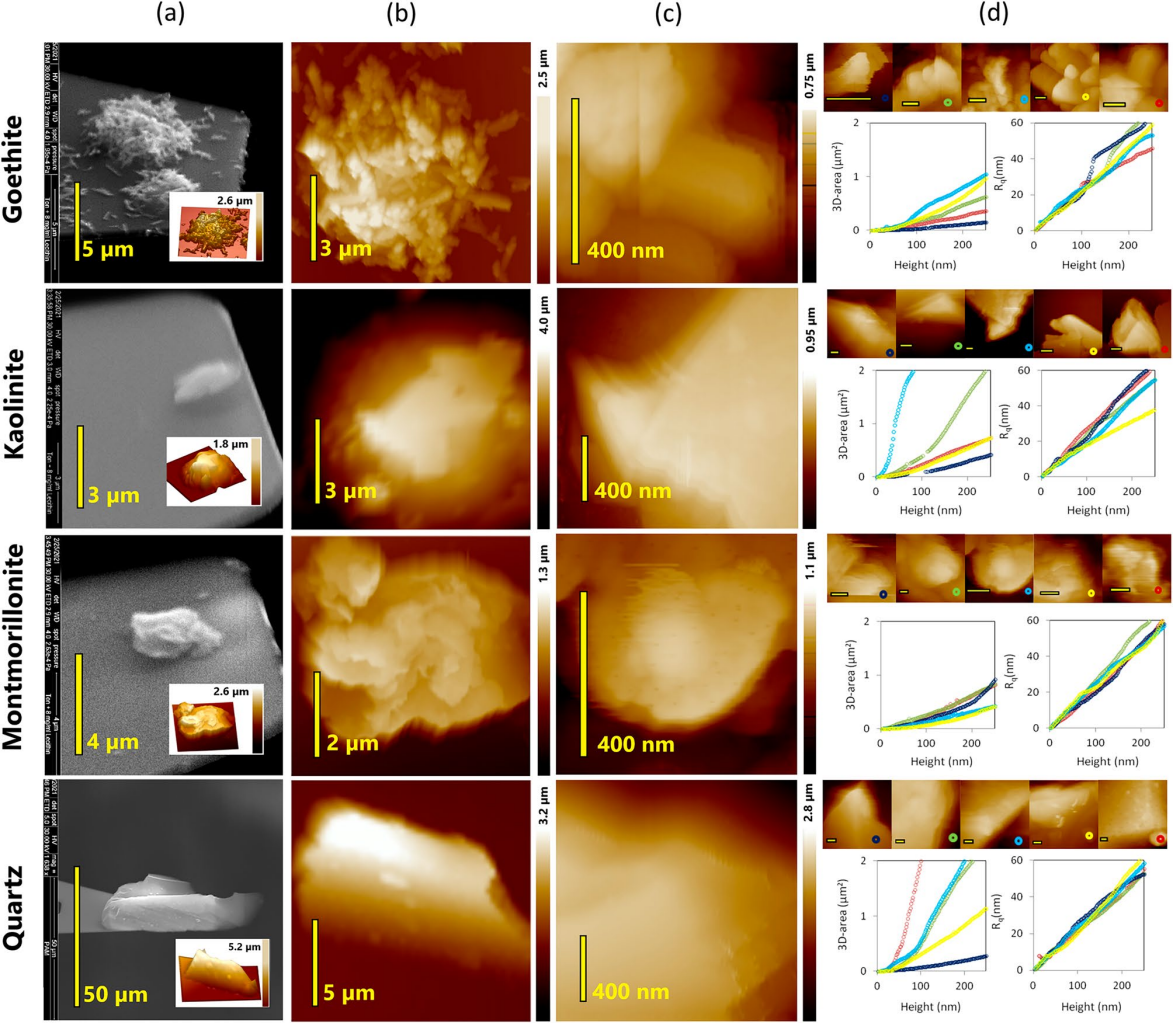
Correlative AFM/ESEM characterizations of tips modified with four different minerals shown for each mineral in one row: (**a**) ESEM pictures of the cantilevers modified with the minerals with insets showing 3D inverse AFM images of the respective modifying mineral, (**b**) closer AFM images of the mineral clusters at the top of the glue spot, (**c**) insets of local areas inside (**b**) with the highest z extension, and (**d**) local AFM maps (scale bar of 200 nm) of the set of 5 probes later used for the cell-mineral interactions and graphs presenting their tip area (left) and R_q_ (right) as a function of height (or deformation depth).

In Fig. 2a, the mineral clusters and quartz particles adhering to the glue of the tipless probe, as imaged by ESEM and AFM, exhibit comparable structures to those presented in Fig. 1. The region of the probes extending most into the z direction (height) as shown in Fig. 2b,c offers a closer look at the “potential” mineral tip when the probe is used to scan bacterial cells. The 3D tip area represented as a function of height for each mineral tip (area-height function) corresponds to the contact area as a function of deformation depth during specific cell-mineral interactions (Fig. 2d and Supplementary chapter S-I.2 with Supplementary Fig. S-I 3 and Supplementary Fig. S-I 4). For the average deformation observed during the cell-mineral interactions, the 3D areas tend to decrease in the order of quartz > kaolinite > montmorillonite > goethite (Supplementary Fig. S-I 5). Both, roughness and 3D area of the particles attached to the probes increase with height, albeit with a narrower range of variation for the 3D area (Fig. 2d). However, although kaolinite and goethite surfaces were expected to be smoother, they did not exhibit lower roughness compared to montmorillonite and quartz surfaces (Fig. 2d). Nevertheless, the tip area-height functions vary considerably among individual modified tips of the same mineral (Fig. 2d). This variation reflects topographical irregularities including orientation, flatness, or aggregated structure of the particles. Despite the fact that the size of the mineral particles or aggregates at the end of the AFM probe is larger than that of a single bacterium, their irregular shapes locally provide nanoscale “tips” for AFM force measurements under low loading forces, and, consequently, low deformation.

### Effects of cell-mineral interactions on cell morphology

**Figure 3:**
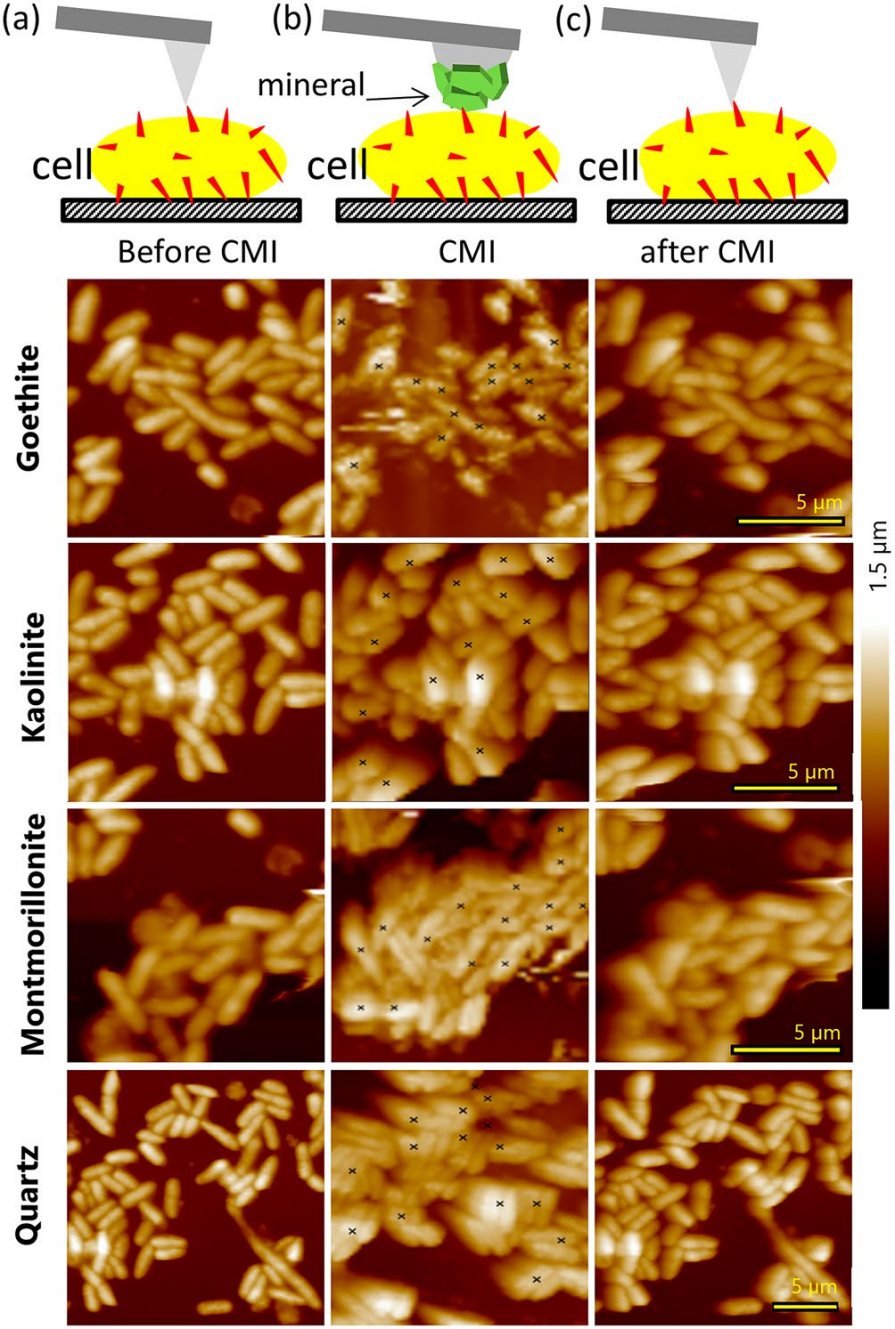
Exemplary images of the same unstressed bacterial cultures in 10 mM KNO_3_ solution for one mineral with the same scale in each row made by (**a**) a sharp tip before the cell-mineral interactions, CMI, (**b**) a mineral modified tip during the CMI with the marks indicating the positions of FD curve acquisition, and (**c**) a sharp tip after the CMI.

To assess the impact of cell-mineral interactions (CMI) on cell integrity, Fig. 3 provides a comparative analysis of height images of the same unstressed *P. fluorescens* cells scanned by (a) standard sharp tip before CMI, (b) a mineral modified tip during CMI and (c) a standard sharp tip after CMI. Generally, for goethite, kaolinite and montmorillonite, similar cell structures are evident in the images obtained before, during and after CMI, underscoring the ability to detect single cells through the proposed modification process. However, in the case of the quartz tip, the interaction does not precisely replicate the shape of the corresponding cells measured by a sharp tip (Supplementary Figs. S-I 7 and 6). It is probable that the large quartz particle made contact with the same cell at different positions acting as multiple tips and producing replicated images of the same cell. Additional images illustrating interactions between both stressed and unstressed cells and several tips for each mineral are provided in Supplementary Fig. S-I 8. These images demonstrate that goethite and in some cases kaolinite tips yielded the best resolution, followed by montmorillonite, while quartz tips occasionally revealed repetitive cell structures. Most importantly, it can be observed that the cell structures remain consistent throughout the cell-mineral interaction, indicating that cell integrity is preserved (Fig. 3a,c).

A more comprehensive interpretation of the measured CMI and the effect of loading force, contact time, and contact area on adhesion forces, adhesion pressure, adhesion efficiency and rupture and adhesion events, is presented in Supplementary Figs. S-I 9 to 13 in chapter S-I.6 and S-I. 7.

### Effect of hypertonic growth conditions of cells for their interactions with minerals

**Figure 4:**
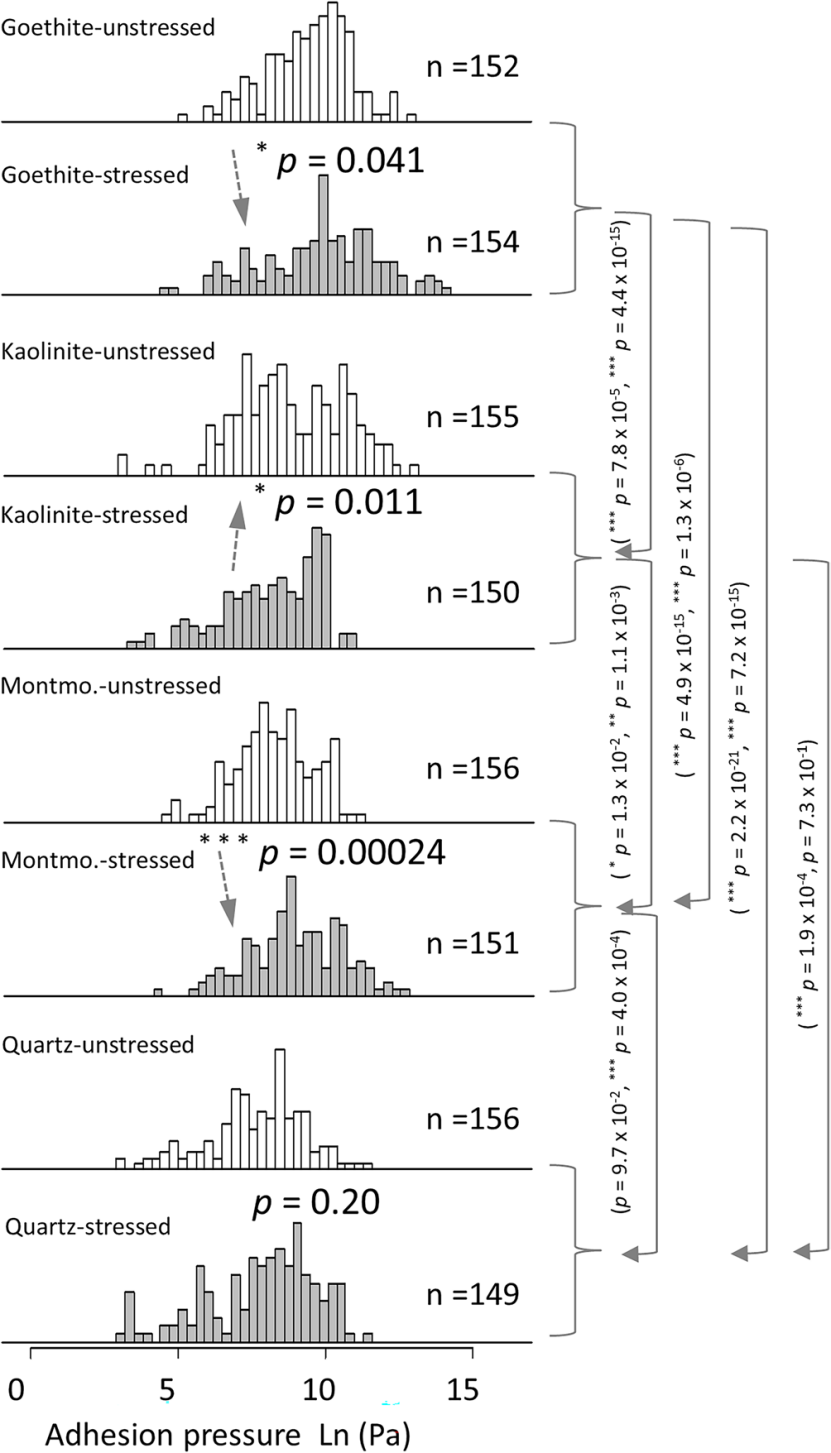
Frequency distributions of adhesion pressures of cell-mineral interactions between cells of two independent unstressed and three independent stressed *P. fluorescens* cultures and five individual functionalized tips for each mineral (Fig. 2d) in 10 mM KNO_3_ solution. A set of ~ 30 FD curves for each tip with 1 s contact time and 5 nN applied force was made. The solid arrows highlight significant differences in adhesion pressures between the minerals (with *p* vales for unstressed and stressed separated by “,”) while the dashed arrows show the effect of stress on adhesion pressure.

To quantitatively compare cell-mineral interactions, the adhesion pressure (P_ad_) obtained from force-distance (FD) curves using five modified tips for each mineral at a fixed loading force (5 nN) on stressed and unstressed cells is presented in Fig. 4. Notably, a considerable variability in the data was observed among tips made from the same interacting materials (Supplementary Fig. S-I 14). The hypertonic stress experienced during the growth phase led to a substantial increase in P_ad_ for *P. fluorescens* cells interacting with montmorillonite rising from 7.4 to 21.1 kPa and with goethite increasing from 30.3 to 94.0 kPa, while P_ad_ decreased when interacting with kaolinite from 24.6 to 7.7 kPa. Conversely, P_ad_ values for *P. fluorescens* cells interacting with quartz were 6.3 kPa and 8.3 kPa for unstressed and stressed cells, respectively, and thus remained relatively unaffected by the growth conditions (Fig. 4). Among the minerals, *P. fluorescens* cells exerted the highest P_ad_ with goethite, followed by kaolinite, and the lowest P_ad_ with montmorillonite and quartz (Fig. 4). These differences in P_ad_ between the minerals were significant, with two exceptions: the difference in P_ad_ between kaolinite and quartz on unstressed cells was offset by the stress-dependent decrease in P_ad_ towards kaolinite. In contrast, P_ad_ values for unstressed cells were similar when interacting with montmorillonite and quartz, whereas stress significantly increased P_ad_ when interacting with montmorillonite.

### Physicochemical properties and surface chemical composition (XPS) of cells and minerals

Table 1 provides physicochemical properties of the minerals and bacteria. Goethite exhibited a positive zeta potential at pH 5.9, while the zeta potentials of the other minerals and the bacteria were negative, with the stressed cells showing slightly more negative potentials than the unstressed cells. The contact angles of the minerals were generally below 45°, indicating complete wettability for quartz and slight water repellency for the other minerals, with goethite having the largest contact angle. The wettability of the minerals was also reflected by the large values (> 37 mJ m^-2^, cf.^30^) of the electron-donor component (*γ*_s_^−^), which exhibited an inverse relationship with the contact angle. In contrast, the non-polar Lifshitz–van der Waals component (*γ*_s_^LW^) was similar for all mineral types. The contact angles of the bacteria were considerably larger than those of the minerals, with a noticeable increase for the cells cultivated under hypertonic stress. Similar to the minerals, *γ*^–^ exhibited an inverse relationship to the contact angle, while *γ*_s_^LW^ remained similar for both stressed and unstressed cells. Both *γ*^–^ and *γ*_s_^LW^ were significantly smaller for bacteria than for the minerals.

**Table 1:** Physicochemical properties of minerals and bacteria: Zeta potential (*ζ*), surface potential (*ψ*)*,* contact angle (CA), and the calculated electron-donor (*γ*_s_^-^), -acceptor (*γ*^+^) and non-polar Lifshitz-van der Waals component of surface free energy (*γ*_s_^LW^).

	*ζ*	*ψ*	*θ*	*γ*_s_^LW^	*γ*_s_^+^	*γ*_s_^−^
	(mV)	(mV)	(°)	(mJ m^−2^)		
Goethite	24.2 ± 4.0	28.6	44 ± 8	43.9	1.3 × 10^–2^	37.3
Kaolinite	− 45.3 ± 1.8	− 53.5	24 ± 2	43.1	2.3 × 10^–3^	58.8
Montmorillonite	− 35.5 ± 2.1	− 41.9	18 ± 3	43.7	5.2 × 10^–4^	63.3
Quartz	− 36.7 ± 4.5	− 43.3	0 ± 0	44.4	1.5 × 10^–2^	66.1
*P. fluorescens* (unstressed)^a^	− 10.6 ± 2.4	− 12.5	67 ± 5	35.8	1.7 × 10^–1^	17.0
*P. fluorescens* (stressed)^a^	− 12.3 ± 1.4	− 14.5	93 ± 2	35.0	2.7 × 10^–1^	3.4

Error margins indicate one standard deviation (zeta potential: *n* = 10; contact angle: *n* = 9).

*ζ*: zeta potential, *ψ*: surface potential, *θ*: solid–water contact angle, *γ*_**s**_: solid surface free energy. Meaning of superscripts: ‘LW’: Lifshitz–van der Waals, ‘+’: electron acceptor, ‘–’: electron donor component of surface free energy, respectively.

^a^Data from Karagulyan et al.^33^

The surface chemical composition of the minerals corresponded to their general chemical composition (Supplementary Fig. S-I 15). Oxygen (O) and silicon (Si) dominated the surface chemical composition of phyllosilicates and quartz, with a notable contribution of carbon (C) at ~ 4–5 at.%. Kaolinite and montmorillonite exhibited larger proportions of aluminum (Al) and traces of iron (Fe), potassium (K), calcium (Ca) and magnesium (Mg), each accounting for less than 1 at.%. For quartz, traces of sodium (Na) and zinc (Zn), each below 0.3 at.%, were detectable. The surface chemical composition of goethite was primarily characterized by O and Fe, but it also featured a substantial amount of C at 14.4 at.% along with traces of chromium (Cr) and sulfur (S) at less than 0.7 at.%. The elevated proportion of surface C can be attributed to C components adsorbed from the gas phase, commonly referred to as ‘adventitious carbon’^31^.

### Comparison between AFM measurements, XDLVO calculations and work of adhesion

Theoretical energy profiles illustrating the interaction between bacterial cells and mineral modified tips, calculated using the XDLVO theory based on the values provided in Table 1, are presented in Supplementary Fig. S-I 16 and Supplementary Fig. S-I 17. Notably, for kaolinite, montmorillonite and quartz, the impact of stress on cell–mineral interaction was evident. While the energy profiles for unstressed cells indicate strong repulsive interactions with these minerals, the profiles for stressed cells featured energy barriers and the presence of primary minima at short separation distances, signifying attractive interactions. In the case of goethite, exclusively attractive interactions were indicated for both unstressed and stressed cells, but with deeper primary minima (indicating stronger attractive interactions) observed for stressed cells. The generally attractive interactions between the cells and goethite can be primarily attributed to the positive surface charge of goethite (Table 1), leading to electrostatic attraction with the negatively charged cells.

Table 2 provides a qualitative comparison of the effect of growth under hypertonic stress on cell-mineral adhesion pressures (P_ad_) measured by AFM and interaction forces calculated based on the XDLVO. The most favorable qualitative agreement between AFM measurements and the predictions based on the XDLVO theory was observed for the goethite-modified tips, with only one out of five tips displaying a trend opposite to what was expected from the XDLVO theory. This was followed by montmorillonite and quartz where two tips exhibited deviating interactions. However, for kaolinite, only one tip displayed agreement with the XDLVO theoretical energy profiles while the other four demonstrated no agreement or even contradictory interactions. Notably, with an aspect ratio of approximately 2.3, the kaolinite tips were on average 1.7, 1.6 and 1.4 times more elongated than montmorillonite, goethite and quartz tips, respectively (Supplementary Fig. S-I 18). This implies that the underlying assumption of our XDLVO calculations, which consider a spherical tip facing a planar cell surface, is best satisfied for montmorillonite and least satisfied for kaolinite.

**Table 2:**
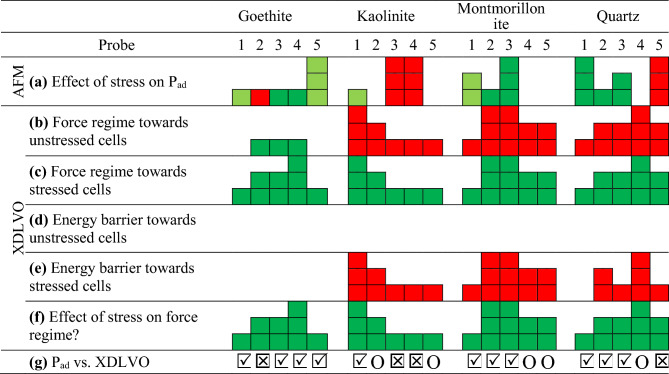
Qualitative comparison of the effect of stress on the cell-mineral interactions between energy profiles calculated by the XDLVO theory from values presented in Table 1 and FD curves obtained by AFM using 5 mineral modified probes for each mineral: (a) Increased (green) or decreased (red) mean adhesion pressures (P_ad_) upon stress, XDLVO based calculated repulsive (red) and attractive (green) forces towards (b) unstressed and (c) stressed cells, XDLVO based calculated energy barriers towards (d) unstressed and (e) stressed cells, (f) absolute shift of the attractive forces (goethite) or repulsive forces (other minerals) of unstressed cells towards more attractive (negative) forces on stressed cells, and (g) agreement ( ), disagreement (i.e., p > 0.1, O), or contradiction ( )) of the response to stress between measured and calculated values. More fields indicate higher effects or size of the respective values and darker colors in (a) indicate p < 0.05, lighter 0.5 < p < 0.1 and no color p > 0.1.

**Figure 5:**
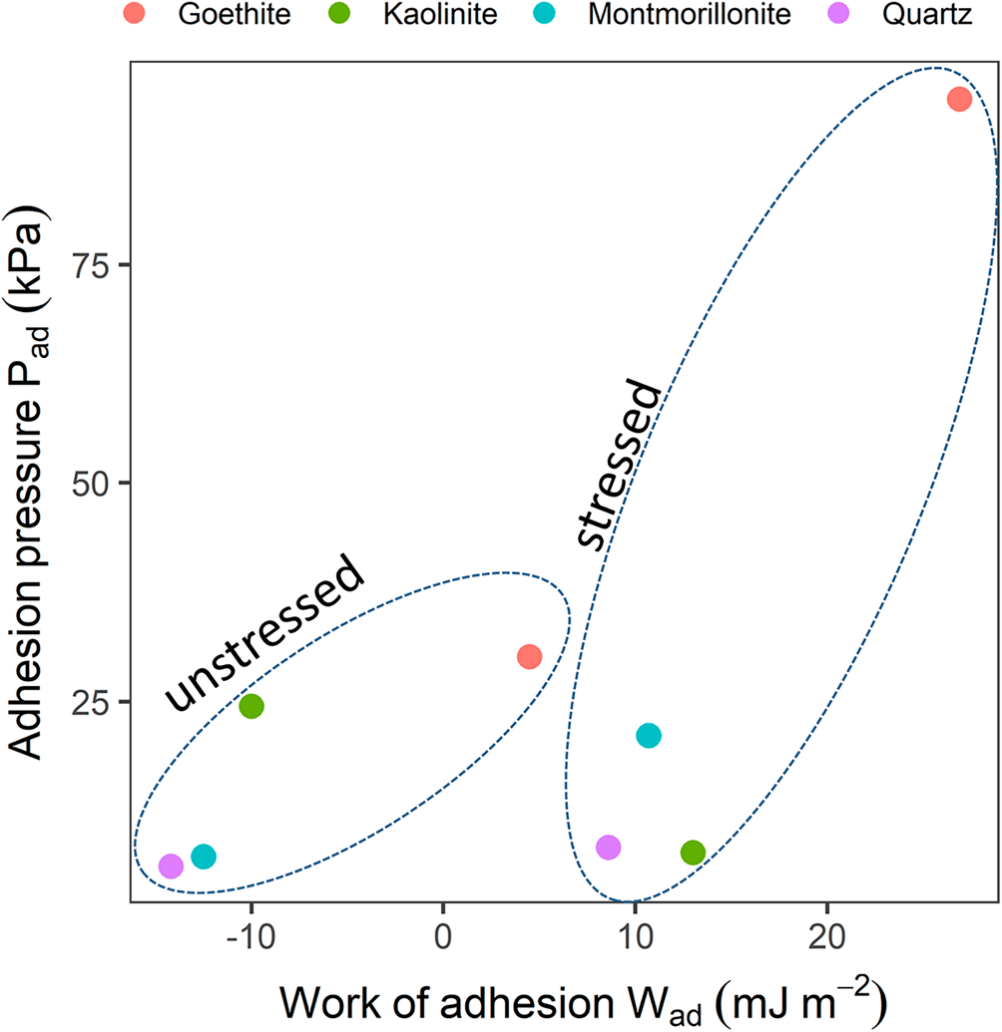
The work of adhesion was calculated from CA data as described in Traini et al.^63^ (Eq. 8). Adhesion pressure values are the mean of the medians extracted from the boxplots (Supplementary Fig. S-I 14) and thus just approximate values to show the general relationship. In contrast to the energy profiles, the negative values found for the CMI with unstressed cells indicate repulsive interactions.

Finally, the work of adhesion (W_ad_) between stressed and unstressed cells and the four minerals calculated from their contact angles exhibits a linear increase with rising P_ad_ for goethite, montmorillonite and quartz. Generally, the values are higher and the slopes are steeper for stressed cells compared to unstressed cells (Fig. 5). Notably, for kaolinite P_ad_ was lower while W_ad_ was higher when interacting with stressed cells compared to unstressed cells. The calculated W_ad_ values were positive for the interactions of stressed cells with all of the minerals under study, suggesting attraction in all cases, although to different extents depending on the mineral. In contrast, the calculated W_ad_ values for unstressed cells in interactions with kaolinite, montmorillonite and quartz are negative, indicating repulsive interactions. Conversely, interactions with goethite were attractive for unstressed cells, yielding positive W_ad_ values.

## Discussion

Contrary to the often reported jump-to-contact events^4^ and the prominent role of electrostatic forces in cell-mineral adhesion^33–35^, we did neither detect attractive nor repulsive ***forces during single cell-mineral interactions (CMI)***. This finding does not support our hypothesis **H1** and suggests that almost no long-range electrostatic forces are involved in the single cell-mineral interactions. This is also in contrast to the calculated energy profiles of goethite, which indicate attractive electrostatic interactions with both unstressed and stressed cell types due to opposite surface charges. One possible explanation could be that repulsive interactions stemming from the positive charges of the poly-L-lysine coating underneath and between the cells may have compensated for the attractive interactions towards the cells. However, since we observed no differences in long-range electrostatic forces for positively or negatively charged minerals, we suggest that the charge of the poly-L-lysine coating has only a negligible effect. Probably, the single cell resolution and the small cell-mineral interaction area in our study rendered the magnitude of the electrostatic forces much smaller than in other studies where a higher number of cells attached to a tipless probe approached a flat goethite surface^4^. Approach FD curves measured by sharp functionalized AFM tips (–COOH, –NH_2_, –CH_3_) on *Pseudomonas fluorescens* displayed a very flat profile and did not show indications of long-range forces^9^. This leads to the conclusion that bacterial adhesion exhibited negligible charge effects, although some levels of repulsion in the form of an energy barrier or van der Waals jump-in interactions beyond the sensitivity of the cantilevers could not be excluded. This suggests that in our observation, it is not the long-range electrostatic interactions^18^ but rather specific short-range forces that play the predominant role in line with recent findings^36–38^. Such specific interactions can be attributed to hydrophobic interactions and hydrogen bonds^35,38,39^. This is further supported by the contact time dependence of the adhesion forces. Long-range nonspecific interactions act instantaneously during the approach to the sample surface and have little^40^ or no^38^ dependence on the contact time. In contrast, when the cell-mineral contact time is extended, more hydrogen bonds may form, leading to higher adhesion forces until saturation is reached after a few seconds (e.g., 4 s)^4^, when all potential bond partners have met. Additionally, or alternatively, bond strengthening during contact time could be due to a rearrangement of non-polar molecule moieties towards the mineral surfaces with low wettability, away from the water interface. The significant decrease in average adhesion and rupture forces, along with a decrease in the frequency of rupture events with decreasing tip-sample contact time, provides evidence that the adhesion forces predominantly originate from specific forces, especially hydrogen bonding^38^. Our choice of a 1-s contact time followed by a 0-s contact is suitable for studying the mechanisms of bond strengthening and inferring the forces that drive initial cell attachment to the mineral phase. Nevertheless, for research purposes focused on studying the viscoelastic properties of cells^16^ or determining the time required for cell-mineral adhesion to fully strengthen^4^, an exploration of cell adhesion at incrementally increasing time intervals is required.

The occurrence of multiple rupture events can be attributed to the stretching of organic molecules (e.g., membrane molecules or extracellular polymeric substances) that adsorb to the mineral tips during single CMI and gradually desorb during tip retraction^4^ (Supplementary Fig. S-I 19). The unbinding forces, observed in the range of ~ 200–700 pN for the various CMI scenarios in this study, suggest that several bonds were broken simultaneously, as a single hydrogen bond rupture force typically occurs at ~ 10 pN^2^. The presence of 10 mM KNO_3_ during the experiments may have amplified this effect by screening the charges and reducing the repulsive tip-sample steric forces. Consequently, this results in a more rigid and compacted polymer layer and higher adhesion forces compared to experiments with lower or no salt concentration^41^. It is plausible that during tip retraction the stretching of molecules is accompanied to some extend by the stretching of the cell membrane until the point when the restoring force of the cantilever exceeds the unbinding forces exerted by the group of molecules adsorbed to the mineral tip.

The positive correlation between contact areas and adhesion forces (Supplementary Fig. S-I 9) lends support to the validity of the approach of using adhesion pressure^16^ (adhesion force per contact area) for comparing measurements obtained with different mineral probes of varying contact areas. Data on adhesion pressure^16^ are not yet commonly reported but significantly enhance the comparability of single CMI results among minerals with different properties, although it is worth noting that the influence of the irregular shapes of the mineral tips on single CMI cannot be entirely ruled out. Nonetheless, the intertwined effects of chemical and morphological heterogeneity are inherent in nature and thus constitute a part of real CMI scenarios, such as those encountered in soil. Our findings concerning the ***impact of mineral properties*** on adhesion pressures align with a study on Gram-negative *Pseudomonas putida* adsorption onto clay minerals^33^, where the quantity of adsorbed cells decreased in the order of goethite > kaolinite > montmorillonite. This observation is further corroborated by an examination of bacterial-mineral suspensions which reveals a distinct pattern^4^. Goethite formed strong and closely bonded connections with bacterial surfaces, while kaolinite also exhibited good adhesion to these cells, although not as robust as goethite. On the other hand, there were hardly any aggregates of bacterial cells found with montmorillonite, indicating a relatively weaker adhesion of this mineral to the cells^4^. In the case of goethite, which had the highest contact angle, hydrophobic interactions, in addition to hydrogen bonds seemed to govern the CMI. This includes interactions between the mineral surface with adsorbed organic impurities and hydrophobic proteins on the cell surface^9^. The favorable adhesion of cells to goethite is consistent with the absence of an energy barrier in the interaction energy profiles. The hydrophilic nature of the quartz surfaces, characterized by the lowest contact angle, facilitates their rewetting during separation from the cell surface. This likely led to a lower single cell-mineral adhesion of quartz compared to kaolinite and montmorillonite which have higher contact angles. In line with other studies^42^, we found that a higher affinity to adsorb organic impurities from the air (adventitious carbon^43^) correlated with a higher contact angle and a greater affinity for other less polar molecules on the cell surface, particularly in the presence of a polar aqueous medium. This may explain the positive correlation between the work of adhesion calculated based on the contact angle data and the adhesion pressure detected by AFM at the single cell level, supporting our hypothesis **H2,** which posits an increasing adhesion affinity with decreasing wettability.

In a previous study, it was found that the increase in the hydrophobicity of *P. fluorescens* due to hypertonic stress was mediated by chemical modifications that resulted in an increase in protein coverage (i.e., the number of hydrophobic domains) on the cell surface at the expense of lipopolysaccharides^9^. The ***effect of cell growth conditions*** on the adhesion pressure towards both goethite and montmorillonite as expressed by a stress-induced increase in P_ad_ of the cells, can be attributed to stronger hydrophobic interactions. These interactions occur due to the presence of more or larger hydrophobic domains on the cell surfaces, and this supports our hypothesis **H3**. This observation aligns with an increase in the contact angle of the surfaces of stressed cells^32^ and a significant increase in the γ_s_^LW^/γ_s_^−^ ratio, which has been previously shown to be related with increasing cell adhesion^44^. It is also consistent with the higher calculated work of adhesion between minerals and stressed cells compared to the unstressed cells. Furthermore, the lack of a significant stress-dependent difference in adhesion towards the hydrophilic quartz particles confirms the importance of hydrophobic interactions in adhesion within a polar aqueous medium during tip-sample detachment. However, the adhesion pressure of kaolinite towards stressed cells is unexpectedly lower than towards unstressed cells, contradicting **H3**. This difference may be attributed to the fact that the contact area between kaolinite and the single cells involves smooth and polar mineral surface structures. The relative reduction of hydrophilic zones on the surfaces of stressed cells may have led to a reduced number of hydrogen bonds, which is not compensated for by hydrophobic interactions with the cell proteins. It is possible that the cell proteins embedded within the outer membrane^45^ are less accessible to the smoother kaolinite sheets compared to the rougher surface of the other minerals, resulting in an overall reduction in adhesion compared to unstressed cells. This phenomenon might also apply to quartz, however, due to the large contact area and the potential contact with more than one cell, quartz may be less affected by the heterogeneity of the cell structure. The aggregation of single goethite needles on the glue tips likely results in a more complex contact area compared to the kaolinite sheets, despite the smooth structure of individual goethite needles.

**Figure 6:**
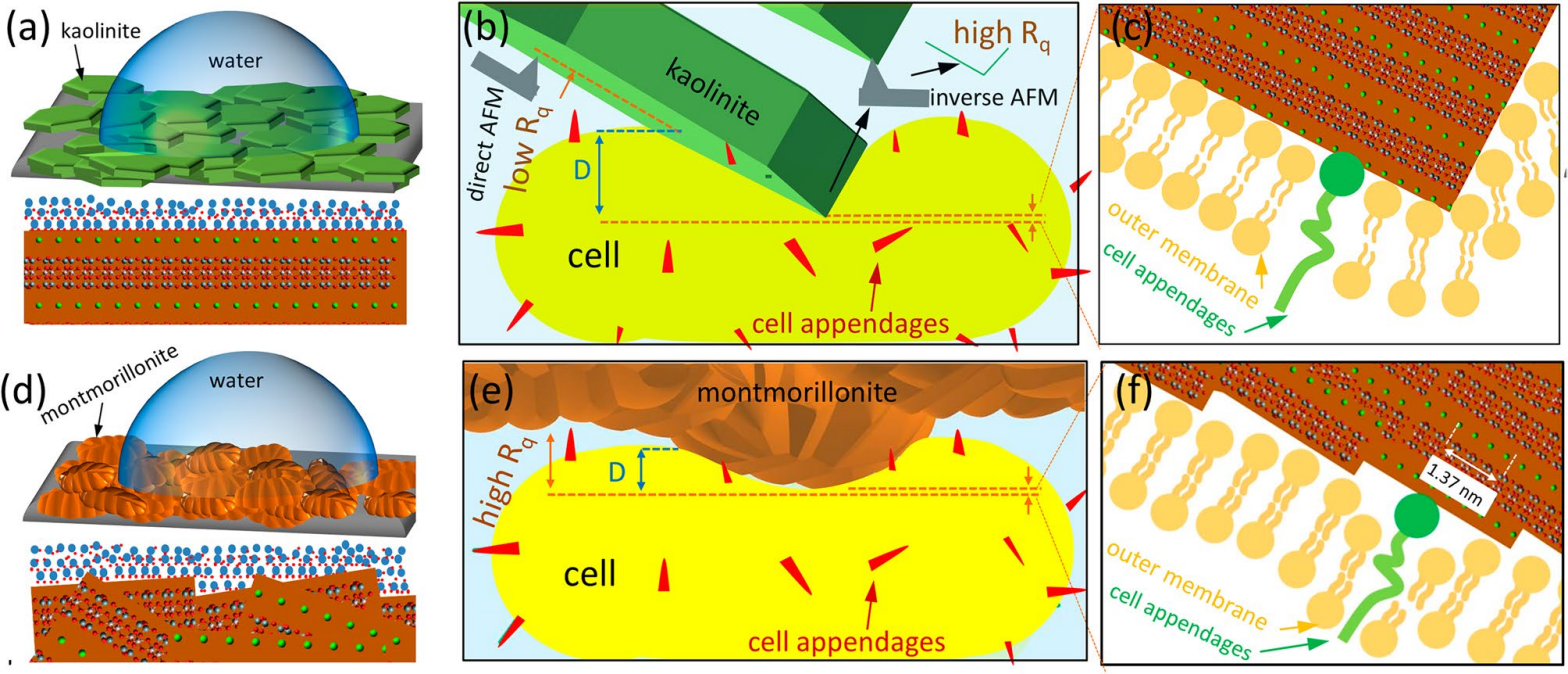
(**a**) sketch of a water drop on a kaolinite surface (top) demonstrating the preferential flat orientation of the particles on the glue surface with the magnification (bottom) showing the interaction between water molecules and the basal planes, (**b**) interaction between a flat kaolinite particle and a planar cell surface with the magnification (**c**) which illustrates how the basal plane with low atomic packing and edge plane with high atomic packing contact the cell surface, (**d**) same as (**a**) for montmorillonite, (**e**) interaction between a porous domain of a montmorillonite particle and a planar cell surface with the magnification (**f**) showing the basal/edge stacking units that get in contact with the cell surface. The crystal pattern is made by Avogadro free software (version 2.0) and it is general for clay minerals. The overlapping basal/edge stacking units of montmorillonite are pure imagination based on our AFM results and the literature^10,27^.

***Deviations from the measured CMI compared to the XDLVO theory*** can be best explained by differences in the geometrical orientation and, consequently, the tip shape of the more or less heterogeneous particle surfaces during single CMI and during physicochemical characterization. In the case of ***kaolinite***, the best agreement of measured adhesion forces with the XDLVO-based predictions were found for the probe with a nearly flat orientation of the kaolinite basal planes, which are also the most exposed when fixed on a flat surface during the contact angle measurements (Fig. 6a). All probes with higher tilt angles of the kaolinite sheets disagreed with the expected theoretical trend, likely because the exposed edge planes form not only more but also stronger hydrogen bonds^26,27,46^ with hydrophilic molecules on the cell surface, resulting in locally higher adhesion forces than at the basal planes (Fig. 6b,c and Supplementary Fig. S-I 18). Although the elongated shape of kaolinite tips makes them unsuitable for directly comparing single CMI determined with AFM with XDLVO predictions, approximating the tip shape to an ideal sphere will only affect the strength of calculated interaction forces^47^. The direction of the forces, i.e. attractive or repulsive, is not affected by an inappropriate geometrical approximation unless the backside of the particle is involved in long-range interactions with the sample surface^48^, which is irrelevant for our study. Thus, the contradiction between single CMI and XDLVO results (Tips 3 and 4, Table 2) cannot be solely attributed to surface irregularities. Instead, it is a combination of both chemical heterogeneity and morphological irregularities that contributes to this disagreement which partially supports hypothesis **H4** that the chemical heterogeneity of kaolinite causes the lowest agreement between AFM measurements and predictions based on XDLVO theory. In this context, it is the different polar site density, rather than the surface charge across different particle planes^4,12,47^, that is responsible for the large heterogeneity of CMI.

In contrast to kaolinite, ***montmorillonite*** possesses porous domains^27^ and, on a small scale, exhibits more irregularities and a significantly higher R_q_ than kaolinite. However, the montmorillonite tips show the most confined and repeatable area-height functions among all studied minerals. This, along with a better agreement of single CMI of montmorillonite compared to kaolinite with XDLVO-based predictions, suggests that montmorillonite tips are more homogeneous over the entire contact area. This homogeneity likely results from the rounder shaped tips with an aspect ratio of 1.4 compared to 2.3 for the kaolinite tips. Probably, the contact area with montmorillonite contains repetitive overlapping textures with clearly visible basal/edge stacking units, leading to a homogeneous mixture of edge and basal planes exposed towards the outer surface^27^. This results in a comparable polar site density of the tips and the layers for the contact angle measurements (Fig. 6d,e,f).

The generally good agreement of the measured adhesion pressure between ***goethite*** and single bacteria with the XDLVO calculations suggests that for goethite, the samples used for contact angle measurement have surfaces more similar to that of the modified tips than for kaolinite. Goethite tends to form aggregates with random particle orientation, exposing both sides and edges of the needles randomly upward. However, there is one exception among the goethite probes that appear to contact the cell surface uniformly across the elongated particle side. For this tip, not only is the aspect ratio significantly larger than for the other tips, reducing the accuracy of the fit to the theoretical predictions, but also the chemistry is different from goethite aggregates, resulting in a contradiction between the measured single CMI and the XDLVO calculations (Supplementary Fig. S-I 18).

The increased adhesion of three ***quartz*** grains towards stressed cells compared to unstressed cells, in agreement with the XDLVO theory, can be explained by the homogeneous chemical structure of this mineral^49^ as well as the sufficient roundness of the tips. However, even though the effect of stress on adhesion is expected to be reproducible among the different quartz tips, two did not follow the theoretical trends. The relatively flat contact region between the two largest quartz particles and cell surfaces may have resulted in multiple contact sites, perhaps even in domains far away from the area of maximum z-extension. This challenges the assumption of single-cell interaction and leads to contradictions with the XDLVO model and an overestimation of the adhesion pressures for quartz. These findings highlight the limitations of our approach for colloidal systems characterized by a significant difference in the size of the interacting materials. However, the underestimation of the contact area does not affect the order of CMI strength among the minerals because quartz exhibited the lowest adhesion and normalization by actual larger contact areas would shift adhesion pressures even lower. Therefore, it is recommended to use a low loading force such as our selected value (5 nN) to achieve single-cell resolution. Otherwise, there is a risk that not only quartz but also the small mineral particles forming clusters at the AFM probe may establish multiple contacts with the cells at higher loads.

## Conclusions

By individually considering the 3D contact area for each cell-substrate pair, this study enabled a direct comparison of cell-mineral interactions through adhesion pressure. Our investigation sheds light on the role of Gram-negative bacterial cells and their cell wall debris in influencing the dynamics of mineral surface properties in soil under changing moisture conditions. From the results, we conclude that the increased hydrophobicity of cells due to hypertonic stress enhances their adhesion towards goethite and montmorillonite. This can lead to the formation of more stable bacterial layers on these minerals, consequently reducing the soil’s surface free energy. Such changes have implications for soil biology and chemistry, as higher adhesion can result in more stable and less easily degradable hydrophobic bacterial layers and thus increase the persistence of soil water repellency. Increased soil water repellency, known to inhibit plant growth and increase bacterial stress, can feed back into the hydrophobizing effect of bacteria. The strong adhesion of bacterial cells to iron oxides underscores the potential role of these minerals in preserving organic matter against degradation and, therefore, long-term soil water repellency. Conversely, a reduced adhesion of stressed cells towards kaolinite may indicate that kaolinite-rich soils are less likely to exhibit long-term water repellency due to bacterial adhesion. However, these results should also be tested with other bacterial strains. The comparison between measured single CMI and XDLVO calculations, based on an idealized sphere-plane geometry, indicates that the interaction between soil matrix and cells is significantly determined by both the type and shape of minerals present. Contradictions in trends between measured and calculated interactions have helped interpret chemical heterogeneity due to differently exposed edges and planes of minerals.

## Methods

### Minerals used for cell–mineral interactions

We employed typical soil minerals, including montmorillonite (Sigma-Aldrich: montmorillonite K 10, no. 69866), kaolinite (Sigma-Aldrich: kaolinite natural, no. 03584), goethite (Bayferrox 920 Z, Lanxess) and quartz (Merck Millipore: quartz fine granular, washed and calcined, no. 1075361000) to investigate cell-mineral interactions. The reported mineral particle sizes for montmorillonite, kaolinite, and goethite were 412 nm^50^, 447 nm^50^, and 580 nm^51^, respectively. Quartz had a particle size of 0.2–0.8 mm according to the technical data sheet.

### Preparation of bacterial cell suspensions

*Pseudomonas fluorescens* DSM 55,090, obtained from the Leibniz Institute DSMZ—German Collection of Microorganisms and Cell Cultures was cultured in 50 ml sterile mineral salt medium containing 7 g Na_2_HPO_4_, 2.8 g KH_2_PO_4_, 0.5 g NaCl, 1 g NH_4_Cl, 0.1 g MgSO_4_ ∙ 7 H_2_O, 0.01 g FeSO_4_ ∙7 H_2_O, 5 mg MnSO_4_ H_2_0, 6.4 mg ZnCl_2_, 1 mg CaCl_2_ ∙ 6 H_2_O, 0.6 mg BaCl_2_, 0.36 mg CuSO_4_ ∙ 7 H_2_O, 0.36 mg CuSO_4_ ∙5 H_2_O, 6.5 mg H_3_BO_3_, 0.01 g EDTA and 146 μl HCl (37%) per liter of distilled water^52^. Additionally, 4 g L^−1^ sodium succinate and 1 g L^−1^ yeast extract were added as carbon sources. Bacterial cells were subjected to osmotic stress by adding 0.5 M NaCl, reducing the water potential of the growth media to − 2.5 MPa. Cell growth was monitored by measuring optical density at 560 nm using a UV/VIS spectrophotometer (Lambda2S, PerkinElmer, Waltham, USA). Cells were harvested during the late exponential growth phase by centrifugation at 11,000*g* for 15 min (Hermle Z383K). They were then resuspended in 2 ml KNO_3_ (10 mM, pH 7.0) and transferred to 2 ml reaction tubes. Each sample was washed twice with 2 ml KNO_3_ followed by 1 min centrifugation at 10,000*g*. The washed biomass was resuspended in 1 ml KNO_3_ and stored at 5 °C until further use.

### Characterization of minerals by AFM

To ensure that the shapes of the mineral particles used as tip modifications were representative, we scanned all minerals in 10 mM KNO_3_ with an AFM system (AFM, Dimension Icon, Bruker Corporation, USA). We used sharp SNL tips (*k* = 0.12 N m^−1^, SNL-10, Bruker, USA) on flat multi-loaded systems^53^ considering 60–150 particles in a total of 55 regions of interest (ROIs). We present an individual image for each mineral to enhance visualization. The root mean roughness (R_q_ = $\sqrt{\Sigma {({Z}_{i})}^{2}/N}$, where Z_i_ is the current Z value, and N is the number of points) was calculated using the roughness function of the NanoScope Analysis software (version 2.0, Bruker) in the Height Sensor channel. For goethite, we estimated the roughness of individual needles or small clusters with clear flat particle orientation by applying a threshold to exclude background data (Supplementary Fig. S-I 1). For the other minerals, we considered only the central areas of the particles for R_q_ estimation to avoid the dilation effect (Supplementary Fig. S-I 6).

### Preparation of cell samples on relocation systems with built-in characterizers

We fixed minerals to the AFM probes and bacterial cells to a flat sample holder and not the other way around^4^. This approach allowed for single cell resolution and eliminated the challenges of attaching single bacterial cells to AFM cantilevers, reducing the risk of potential damage during probe calibration^18^.

Preparation of the sample holders in the form of relocation systems followed a modified protocol of *Abu Quba* et al.^23^. First, cover glasses (hydrolytic class 1, 50 × 24 × 0.13–0.16 mm, VWR, Germany) were cleaned first with acetone (ROTISOLV® Pestilyse® plus ≥ 99.9%, R T, Germany) and immediately before drying with ~ 70% ethanol (ROTIPURAN® ≥ 99.8%, p.a, R T, Germany) for 30 min in a conventional ultrasonic bath. Each cover glass with a small piece of resin (Tempfix, PLANO GmbH, Wetzlar, Germany) was heated up to 130 °C for 30 s to melt the resin and create a flat surface. Then, a piece of freshly cleaved mica was fixed at the resin spot at 80 °C and an extracted and blind tip reconstructed SNL tip was fixed at the resin near the mica sheet at 38 °C. On the back side of the glass slide, a finder grid was fixed with transparent adhesive tape near the glue spot (Supplementary Fig. S-I 20). The shape, size and stiffness of stressed and unstressed *P. fluorescens* cells were found to be comparable when fixed at chemically inactive glue^32^ or poly-L-lysine^9^ surfaces. Therefore, poly-L-lysine (P8920, 166 Sigma-Aldrich, Germany) was used to improve cell fixation in this work. A drop of the latter was pipetted onto the front side exactly over the grid and air dried. The cells were attached by pipetting 1–2 µL of cell suspensions onto the poly-L-lysine spot, air dried for 1 min and rinsed before being covered with a drop of 10 mM KNO_3_ solution in which the AFM measurement was performed. To maintain a constant concentration of 10 mM KNO_3_ during the scanning, we pumped water to the liquid meniscus at a rate equal to the evaporation rate using a custom-built pump which was used in our previous work^9^. The mica surface allowed direct calibration of the deflection sensitivity of the probe in liquid without changing the samples, while the blind tip reconstructed SNL tip served for quality control of the functionalized tips before and after the cell-mineral interactions by repeated inverse imaging^23^.

### Functionalization of tipless cantilevers with minerals

To functionalize the tips, we started by attaching a small amount of Tempfix resin to the end of an SNL probe (*k* = 0.12 N m^−1^, SNL-10, Bruker, USA) following the detailed procedure outlined by *Abu Quba* et al.^23^. Using the AFM XYZ navigation system, we gradually reduced the size of the resin at the tip of the SNL probe by making repeated contacts with a chip (visible as glue spots on the chip in Supplementary Fig. S-I 21) securely fixed to the AFM stage. This chip was heated to ~ 100 °C allowing us to transfer tiny resin spots from the SNL tip to a set of tipless probes (*k* = 0.1 N m^−1^, MLCT-O10, Bruker, USA), also fixed to the heated AFM stage. For the attachment of smaller particles, such as montmorillonite, kaolinite, or goethite to the resin coated tipless cantilevers, we sprinkled these particles over the resin spot and then subjected them to a 30 min heating at 38°C in an oven to enhance the adhesion between the minerals and the resin. Afterwards, the modified probe underwent a thorough rinse with Milli-Q water to remove any loosely bound particles followed by air drying.

In the case of quartz particles, epoxy glue (Pattex Kraft Mix, Henkel AG, Germany) was employed instead of resin. Here, a tipless probe was guided by the AFM navigation system, first into a glue spot at room temperature and then onto a quartz particle. It was allowed to sit for 3 min to ensure secure fixation. Subsequently, the modified probe underwent the same cleaning as described above.

To ensure the integrity of our probes, we conducted checks, both before and after cell-mineral interaction measurements. We utilized AFM inverse imaging and environmental scanning electron microscopy (FEI Quanta 250 ESEM, FEI Company, Hillsboro, United States). Only the results obtained from mineral tips that showed no changes in the modifying minerals after cell-mineral interaction measurements were considered for our analysis.

### Direct cell–mineral interactions

Before commencing the cell-mineral interaction experiments, we determined the spring constant through a thermal tune, and calibrated the deflection sensitivity of each modified probe using force distance curves on integrated mica sheets. We conducted direct cell-mineral interactions using the “Point and Shoot” function in peak force quantitative nanomechanical mapping (PFQNM) mode employing conditions of 10 mM KNO_3_, a 1.03 Hz ramp rate (with forward and reverse velocity of 6 µm s^-1^), a 5 nN force set point, 3 μm ramp size and contact times of 1 and 0 s after reaching the force set point (illustrated in Supplementary Fig. S-I 12). In relocation systems involving stressed and unstressed cells, we selected 5 to 8 ROIs. Within all ROIs we acquired ~ 150 force-distance (FD) curves on the highest points of the cells. To better capture the response of the cell communities, we distributed the FD curves across as many cells as possible in the scanned images, rather than conducting numerous replicates on the same cell. In instances where rupture events occurred, we measured the magnitude and distance of the last event when the tip fully separated from the surface using the markers function of NanoScope software. To assess the effect of enlarging the contact area on adhesion forces, we recorded 10 pull-off curves with one selected probe for each mineral. These curves were measured against both unstressed and stressed cells, as well as against double-sided adhesive tape (Tesa, Hamburg, Germany) with incremental loading forces ranging from 2 to 30 nN (as shown in Supplementary Fig. S-I 13). The contact area and the number of contact points between tip and sample depend on the shape of the modified probe and the applied loading force. While flat^4^ or semi flat^16^ surfaces (e.g., those with a round shape and a large radius) can establish multiple contact points with the sample even with low loading forces like 1 nN^4^ or 10 nN^16^, our specific design involved the introduction of a glue drop at the end of the cantilever, allowing the minerals attached to the glue to provide sharp features. In most cases, a loading force of 5 nN proved sufficient to achieve single cell resolution.

### Quality control of direct cell–mineral adhesion

To avoid artifacts, we rigorously validated the stability of selected bacteria and minerals during cell-mineral interaction. This involved several steps: (1) We examined all mineral-modified tips using ESEM and some were further analyzed using correlative ESEM/AFM method prior to cell-mineral interaction. (2) After cell-mineral interactions, we checked all mineral modified tips with AFM at the built-in characterizer to obtain the tip-area-function. (3) We also inspected all cells with AFM using a sharp tip or the respective mineral tip (if sufficiently sharp), both before and after cell-mineral interaction. For more details please refer to Abu Quba et al.^23^.

The FD curves were recorded with 1-s contact time followed by another curve with 0-s contact time. A decrease in adhesion forces with decreasing contact time served as a direct evidence that no irreversible modifications of the cell ultrastructure had occurred, as descried in previous studies^16,39^.

### Evaluation of the FD curves

We determined the adhesion force using R (R Core Team, 2020) (^9,54^). The FD curve data were exported from the NanoScope software as csv files, and the adhesion force was determined as the minimum force of the baseline corrected retraction curve. Each adhesion force was normalized by the 3D area of the respective tip that interacted with the cell surface at the specific deformation depth, yielding the adhesion pressure^16^ P_ad_ (Supplementary Fig. S-I 4). We also generated 2D sections of the mineral tips at two deformation levels (20 nm and 50 nm) to compare the ratio of the shortest and longest elongation, which helped to describe the shape deviation from the spherical shapes in our XDLVO calculations. We investigated the impact of loading force on adhesion force by calculating the adhesion efficiency η (%) defined as the ratio of the measured adhesion force to the applied loading force^16^.

To identify attractive interactions between the mineral modified-tips and the cell surfaces, we screened the FD curves for jump-to-contact events using the NanoScope Analysis software. In contrast, repulsive forces were identified as a gradual increase in the force curve as the distance to the surface decreased, before reaching the “linear” contact regime.

### Zeta potential measurement and estimation of surface potential

Zeta potential measurements on bacteria and minerals were conducted to estimate their surface potential as a basis for calculating electrostatic interaction free energy. Zeta potential was calculated using Smoluchowski’s equation^55^ based on electrophoretic mobility which was measured through phase analysis light scattering (ZetaPALS, Brookhaven Instruments Corp., Holtsville, USA). Bacterial cells were suspended in a 10 mM KNO_3_ solution, adjusted to pH 6 using 1 M HNO_3_, in a concentration of ~ 10^9^ cells L^–1^. Mineral particles were suspended in a 10 mM KNO_3_ solution adjusted to pH 6 at concentrations ranging from 23 to 40 mg L^–1^ (equivalent to ~ 0.001% by volume). Before measurement, the quartz particles were ground to an appropriate size for zeta potential measurements using an agate mortar, resulting in a mean particle size of ~ 1350 nm based on dynamic light scattering (ZetaPALS). The mean zeta potential was determined from 10 consecutive runs, each comprising 10 cycles.

Surface potential (*ψ*) was estimated from the zeta potential (*ζ*) using Eq. (1)^56^:

1 $$\psi = \zeta \left( {1 + \frac{z}{R}} \right)\exp (\kappa z)$$

where *z* is the distance from the surface to the slipping plane, assumed as 0.5 nm^56^, *R* represents the respective radius of the cells or the mineral particles, and *κ* is the inverse of the double layer thickness (m^−1^). The value of *κ* was calculated using Eq. (2)^57^:

2 $$\kappa^{ - 1} = \sqrt {\frac{{\varepsilon_{{\text{r}}} \varepsilon_{{0}} k_{{\text{B}}} T}}{{2N_{{\text{A}}} e^{2} I}}}$$

where *ε*_r_ is the relative dielectric permittivity of water (80.1 at 20 °C), *ε*_0_ is the vacuum permittivity [8.854 × 10^–12^ C/(Vm)], *k*_B_ is the Boltzmann constant (1.38 × 10^–23^ J K^–1^), *T* is the absolute temperature (K), *N*_A_ is the Avogadro number (6.02 × 10^23^ mol^–1^), *e* is the charge of the electron (1.6 × 10^–19^ C), and *I* is the ionic strength of the KNO_3_ solution (10 mol m^–3^).

### Contact angle measurement and calculation of surface free energy

Contact angles of bacteria and minerals were determined with a contact angle microscope equipped with a video camera (OCA 15, DataPhysics, Filderstadt, Germany). Bacterial cell samples were prepared by filtering cell suspension through cellulose acetate filters (pore size 0.45 mm, NC 45; Whatman) and fixing the air-dried filters on microscopy glass slides. Mineral samples were prepared by gently pressing air-dried mineral particles onto double-sided adhesive tape covering a microscopy glass. Non-adherent particles were removed by tapping the slide until no further material loss was observed. A drop of deionized water (1 µl) was placed on the sample surface immediately after preparation, and the initial water contact angle was measured at both intersections of the drop contour line with the sample surface using automated drop shape analysis with software SCA20 (DataPhysics, Filderstadt, Germany). Mean contact angles were calculated from ten independent measurements. Further details on the method and sample preparation can be found in Bachmann et al.^58^ and Goebel et al.^59^.

Additional contact angle measurements were performed using ethylene glycol and α-bromonaphthalene as testing liquids to calculate the surface free energy components of bacteria and minerals, forming the basis for determining cell–mineral interaction energy. Solid and liquid interfacial properties were linked through the solid–liquid contact angle (*θ*) as expressed by Eq. (3)^60^:

3 $$\left(1+{\text{cos}}\theta \right){\gamma }_{\text{l}}=2\left(\sqrt{{\gamma }_{\text{s}}^{\text{LW}}{\gamma }_{\text{l}}^{\text{LW}}}+\sqrt{{\gamma }_{\text{s}}^{+}{\gamma }_{\text{l}}^{-}}+\sqrt{{\gamma }_{\text{s}}^{-}{\gamma }_{\text{l}}^{+}}\right)$$

where *γ*_l_ is the liquid surface free energy (J m^–2^), and *γ*_s_ is the solid surface free energy (J m^–2^). Superscripts ‘LW’, ‘-’, and ‘ + represent the non-polar Lifshitz–van der Waals component, the electron-donor (base) component, and the electron-acceptor (acid) component, respectively. The three unknown variables in Eq. (3), *γ*_s_^LW^, *γ*_s_^–^, *γ*_s_^+^, were determined by solving a system of three independent linear equations, using the mean contact angles obtained with deionized water, ethylene glycol, and α-bromonaphthalene along with the respective surface free energy components of the liquids^60^.

### Calculation of the cell–mineral interaction energy profiles

Energy profiles of the interaction between bacterial cells and AFM tips functionalized with mineral particles were determined by calculating the total interaction free energy, Δ*G*_132_^TOT^, between the mineral (1), and the bacterial cell (2) in aqueous solution (3). This calculation considered electrostatic, Δ*G*_132_^EL^, Lifshitz–van der Waals, Δ*G*_132_^LW^, and Lewis acid–base, Δ*G*_132_^AB^, interaction free energies as a function of separation distance, as described by Eq. (4).

4 $$\Delta G(h)_{132}^{{{\text{TOT}}}} = \Delta G(h)_{132}^{{{\text{EL}}}} + \Delta G(h)_{132}^{{{\text{LW}}}} + \Delta G(h)_{132}^{{{\text{AB}}}}$$

Mineral functionalized tips used for measuring adhesion forces on bacterial cells were small compared to the cells. Therefore, we approximated the cell surface as planar geometry, and the mineral functionalized tip as a sphere with a diameter estimated from the contact radius individually determined for each tip. Energy profiles were determined for each combination of minerals and cells by explicitly considering the specific contact radius of each functionalized tip.

The electrostatic interaction free energy, Δ*G*_132_^EL^ (J), was calculated using Eq. (5)^61^:

5 $$\Delta G(h)_{132}^{{{\text{EL}}}} = \pi R_{{\text{m}}} \varepsilon_{{\text{r}}} \varepsilon_{{0}} (\psi_{1}^{2} + \psi_{2}^{2} )\left\{ {\frac{{2\psi_{1} \psi_{2} }}{{\psi_{1}^{2} + \psi_{2}^{2} }}\ln \left[ {\frac{1 + \exp ( - \kappa h)}{{1 - \exp ( - \kappa h)}}} \right] + \ln [1 - \exp ( - 2\kappa h)]} \right\}$$

where *R*_m_ is the contact radius of the mineral tip (m), *h* is the separation distance between the mineral tip and the cell (m), and *ψ*_1_ and *ψ*_2_ are the surface potentials of the minerals and cells (V), respectively. *R*_m_ was determined for each functionalized tip based on the average deformation of the bacterial cell surface.

The Lifshitz–van der Waals interaction free energy component, Δ*G*_132_^LW^ (J), was calculated by Eq. (6)^62^:

6 $$\Delta G(h)_{132}^{{{\text{LW}}}} = - 4\pi R_{{\text{m}}} \frac{{h_{0}^{2} }}{h}\left( {\sqrt {\gamma_{3}^{{{\text{LW}}}} } - \sqrt {\gamma_{2}^{{{\text{LW}}}} } } \right)\left( {\sqrt {\gamma_{3}^{{{\text{LW}}}} } - \sqrt {\gamma_{1}^{{{\text{LW}}}} } } \right)$$

where *h*_0_ is the minimum equilibrium distance of 0.157 nm where physical contact occurs.

The Lewis acid–base interaction free energy component Δ*G*_132_^AB^ (J) was calculated by Eq. (7)^62^:

7 $$\begin{aligned} \Delta G(h)_{132}^{{{\text{AB}}}} & = 4\pi R_{{\text{c}}} \lambda \exp \left( {\frac{{h_{0} - h}}{\lambda }} \right)\left[ {\sqrt {\gamma_{3}^{ + } } \left( {\sqrt {\gamma_{1}^{ - } } + \sqrt {\gamma_{2}^{ - } } - \sqrt {\gamma_{3}^{ - } } } \right)} \right. \\ & \quad + \left. {\sqrt {\gamma_{3}^{ - } } \left( {\sqrt {\gamma_{1}^{ + } } + \sqrt {\gamma_{2}^{ + } } - \sqrt {\gamma_{3}^{ + } } } \right) - \sqrt {\gamma_{1}^{ + } \gamma_{2}^{ - } } - \sqrt {\gamma_{1}^{ - } \gamma_{2}^{ + } } } \right] \\ \end{aligned}$$

where *λ* is the decay length of water (0.6 nm^62^).

The work of adhesion (*W*_ad_) between minerals and bacterial cells was calculated using Eq. (8)^62,63^:

8 $$W_{{{\text{ad}}}} = - \Delta G_{132} = - 2\left[ \begin{gathered} \sqrt {\gamma_{1}^{{{\text{LW}}}} \gamma_{3}^{{{\text{LW}}}} } + \sqrt {\gamma_{2}^{{{\text{LW}}}} \gamma_{3}^{{{\text{LW}}}} } - \sqrt {\gamma_{1}^{{{\text{LW}}}} \gamma_{2}^{{{\text{LW}}}} } - \gamma_{3}^{{{\text{LW}}}} \hfill \\ \quad + \sqrt {\gamma_{3}^{ + } } \left( {\sqrt {\gamma_{1}^{ - } } + \sqrt {\gamma_{2}^{ - } } - \sqrt {\gamma_{3}^{ - } } } \right) + \gamma_{3}^{ - } \left( {\sqrt {\gamma_{1}^{ + } } + \sqrt {\gamma_{2}^{ + } } - \sqrt {\gamma_{3}^{ + } } } \right) \hfill \\ \quad - \sqrt {\gamma_{1}^{ + } \gamma_{2}^{ - } } - \sqrt {\gamma_{1}^{ - } \gamma_{2}^{ + } } \hfill \\ \end{gathered} \right]$$

### X-ray photoelectron spectroscopy

The surface elemental composition of mineral particles was analyzed using X-ray photoelectron spectroscopy (XPS) with an Axis Ultra DLD instrument (Kratos Analytical, Manchester, UK) equipped with monochromatic AlKα radiation (1486.6 eV; emission current: 20 mA, voltage: 6 kV). Samples were prepared by affixing air-dried minerals onto a bar (sample area: 50 mm^2^) using indium foil (Plano GmbH, Wetzlar, Germany). Survey spectra were obtained in the binding energy range of 1200–0 eV (with a 1 eV resolution) under a pressure of 4 × 10^7^ Pa. The measurements utilized a pass energy of 160 eV, a dwell time of 500 ms, and comprised three sweeps per measurement cycle at a take-off angle of 0°. For each sample, three spectra were recorded at different locations (spot size: 300 × 700 µm). After charge correction for the Si 2p peak of quartz (103 eV), the spectra were analyzed using Vision 2 software (Kratos Analytical, Manchester, UK). Surface elemental composition was quantified in terms of atom percentage (at.-%) using the relative sensitivity factors incorporated in the software. For additional details regarding the fitting procedure, please refer to Woche et al.^58^.

### Statistics

Using R^64^, we assessed the adhesion pressure for each mineral and stress level (N = 149–156) for normality through the Shapiro–Wilk-Test (shapiro.test) and checked for variance homogeneity using the Levene-Test (leveneTest). As the results did not indicate homogeneity, we conducted Wilcoxon-Rank-Sum tests (wilcox.test) to identify significant differences in adhesion pressure between stressed and unstressed bacterial cells when interacting with the four different minerals. To detect significant differences in adhesion pressure among different minerals when interacting with cells of the same stress level, we employed pairwise Wilcoxon-Rank-Sum tests (pairwise.wilcox.test). Specific results are presented in chapter S-I.15 and in Fig. 4.

### Supplementary Information

Supplementary Information.

## Data Availability

The datasets generated and/or analyzed during the current study are available in the OSF repository [https://osf.io/6guwd].
