# Supplementary material for: Hypertonic stress induced changes of Pseudomonas fluorescens adhesion towards soil minerals studied by AFM
Source: Sci Rep. 2023 Oct 10;13:17146. doi: 10.1038/s41598-023-44256-7 (PMC10564757; doi:10.1038/s41598-023-44256-7)
Supplement: Supplementary file 1 — Supplementary Information. [file 41598_2023_44256_MOESM1_ESM.pdf]

Supplementary Information to

"Hypertonic stress induced changes of *Pseudomonas fluorescens* adhesion towards soil minerals studied by AFM"

Abd Alaziz Abu Quba[1]; Marc-Oliver Goebel[2]; Mariam Karagulyan[3]; Anja Miltner[3]; Matthias Kästner[3]; Jörg Bachmann[2]; Gabriele E. Schaumann[1]; Dörte Diehl[1]

[1]*Institute for Environmental Sciences, University of Kaiserslautern-Landau (RPTU), Landau, Germany*

[2]*Institute of Soil Science, Leibniz Universität Hannover, Hannover, Germany*

[3]*Helmholtz Centre for Environmental Research – UFZ, Department of Environmental Biotechnology, Leipzig, Germany*

* *Corresponding author: diehl@uni-landau.de*

Content

**S-I.1**	**Characterization of the minerals and their aggregates ……………………..**	**3**
	Fig. S-I 1 ………………………………………………………………………..	4
	Fig. S-I 2 ………………………………………………………………………..	5
**S-I.2**	**Creating the tip area-height functions ………………………………………..**	**5**
	Fig. S-I 3 ………………………………………………………………………..	6
	Fig. S-I 4 ………………………………………………………………………..	7
**S-I.3**	**3D area of CMI ……………………………………………………………….**	**8**
	Fig. S-I 5 ………………………………………………………………………..	8
**S-I.4**	**Deconvolution of irregular shaped particles ……………………………….**	**9**
	Fig. S-I 6 ………………………………………………………………………..	9
	Fig. S-I 7 ………………………………………………………………………..	9
**S-I.5**	**Cell-mineral interaction ………………………………………………………**	**10**
	Fig. S-I 8 ………………………………………………………………………..	11
**S-I.6**	**Adhesion force, deformation and surface area versus loading force**	**12**
	Fig. S-I 9 ………………………………………………………………………..	12
	Fig. S-I 10 ……………………………………………………………………….	13
**S-I.7**	**Effect of contact time …………………………………………………………...**	**14**
	Fig. S-I 11 ……………………………………………………………………….	14
	Fig. S-I 12 ……………………………………………………………………….	15
	Fig. S-I 13 ……………………………………………………………………….	16
**S-I.8**	**Box plots of Pad ………………………………………………………………**	**17**
	Fig. S-I 14 ……………………………………………………………………….	17
**S-I.9**	**Surface chemical composition ………………………………………………**	**18**
	Fig. S-I 15 ……………………………………………………………………….	18
**S-I.10**	**Cell-mineral interaction energy profiles ……………………………………**	**19**
	Fig. S-I 16 ……………………………………………………………………….	20
	Fig. S-I 17 ……………………………………………………………………….	21
**S-I.11**	**Tilt of the mineral particles …………………………………………………**	**22**
	Fig. S-I 18 ……………………………………………………………………….	22
**S-I.12**	**Unbinding force ………………………………………………………………**	**23**
	Fig. S-I 19 ……………………………………………………………………….	23
**S-I.13**	**Relocation system ……………………………………………………………**	**23**
	Fig. S-I 20 ……………………………………………………………………….	23
**S-I.14**	**Functionalization of tipless cantilevers with glue ………………………….**	**24**
	Fig. S-I 21 ……………………………………………………………………….	24
**S-I.15**	**Results from statistical tests ………………………………………………….**	**25**
**S-I.16**	**References ……………………………………………………………………**	**26**

S-I.1Characterization of the minerals and their aggregates

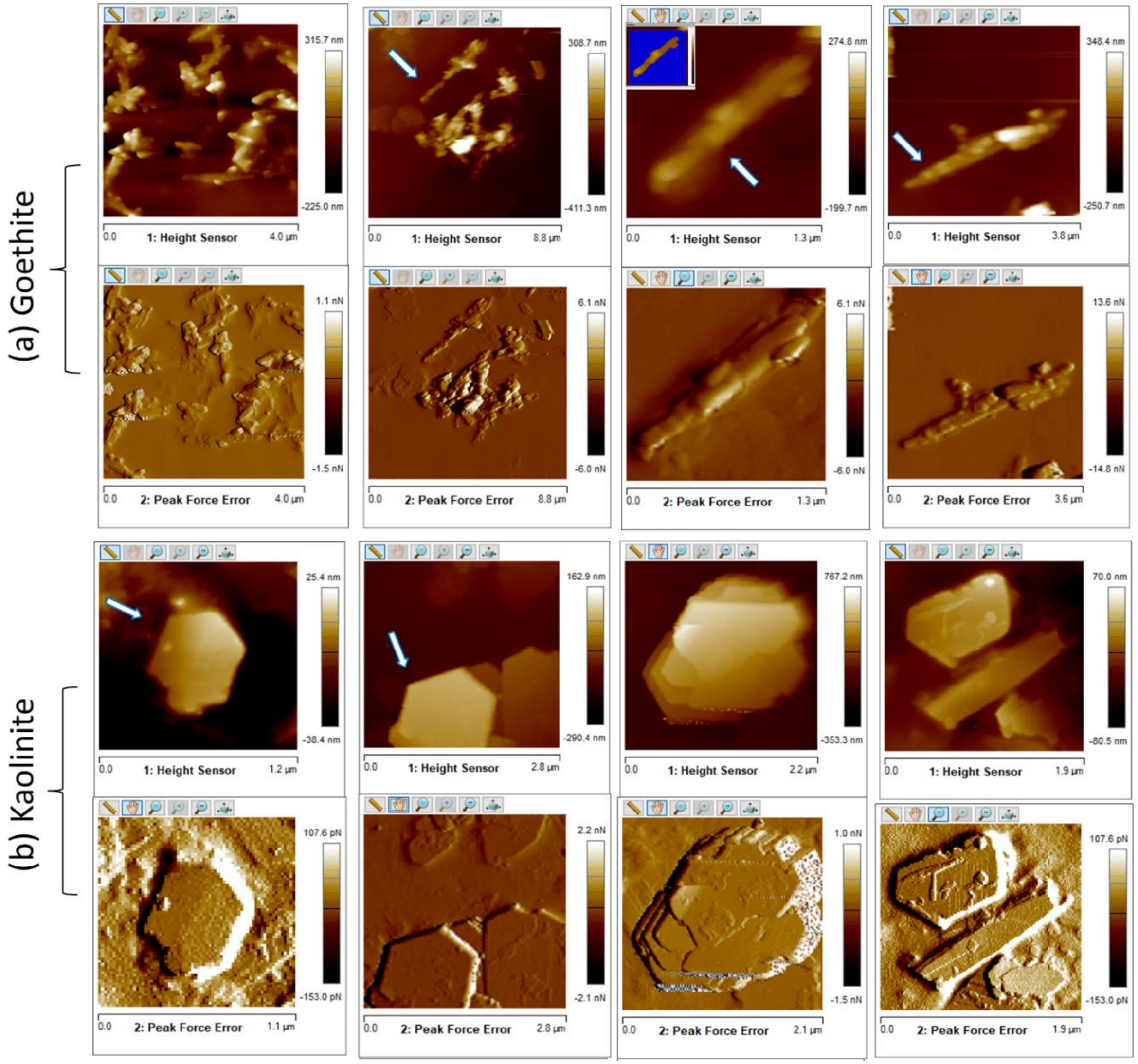

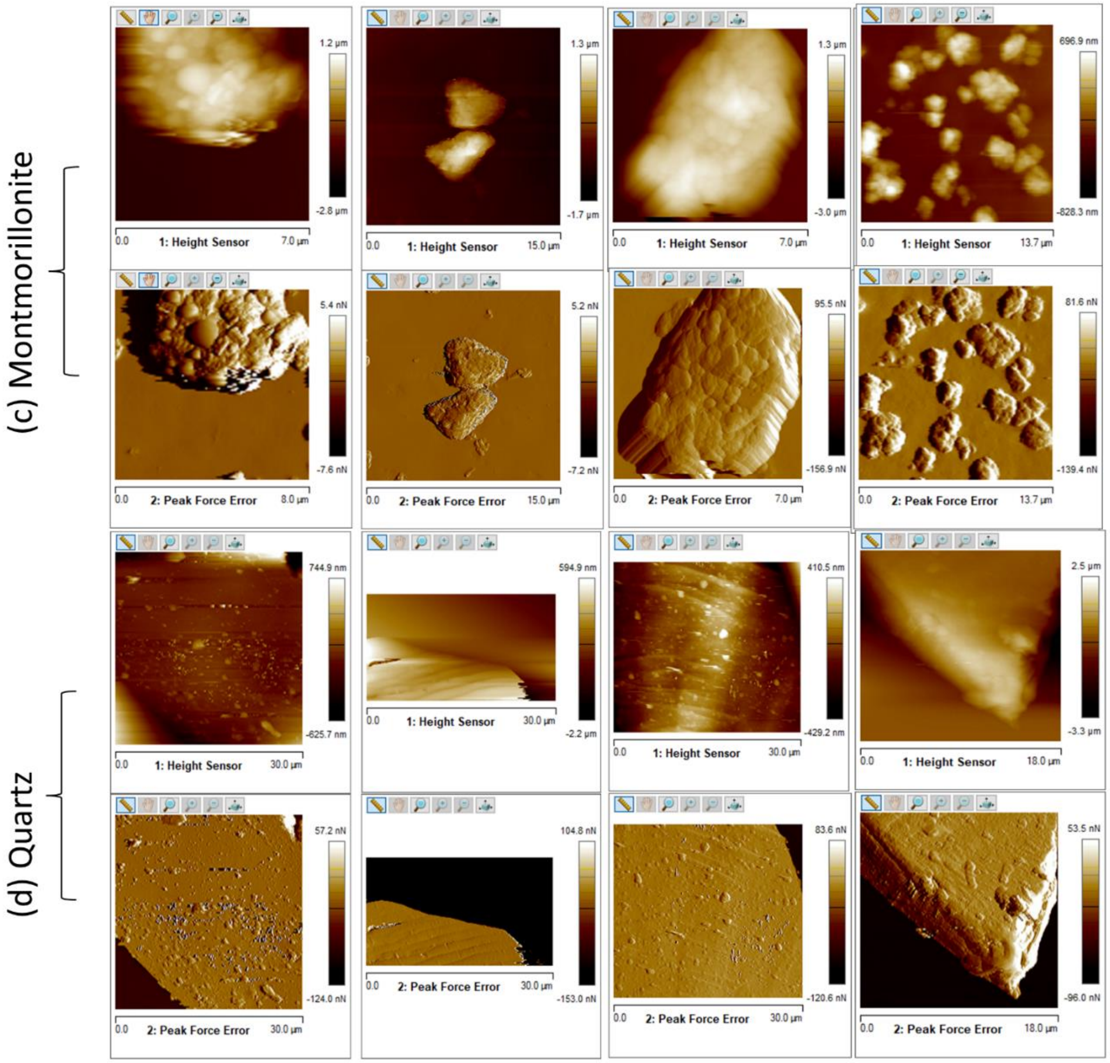

Fig. S-I 1 AFM Height and Peak Force Error maps of **(a**) goethite, **(b)** kaolinite, **(c)** montmorillonite and **(d)** quartz particles obtained by sharp tips in KNO_3 solution. Arrows indicate individual goethite particles or pseudohexagonal kaolinite particles. The inset in (a) shows the applied height threshold to exclude background data for R_q estimation (blue frame).

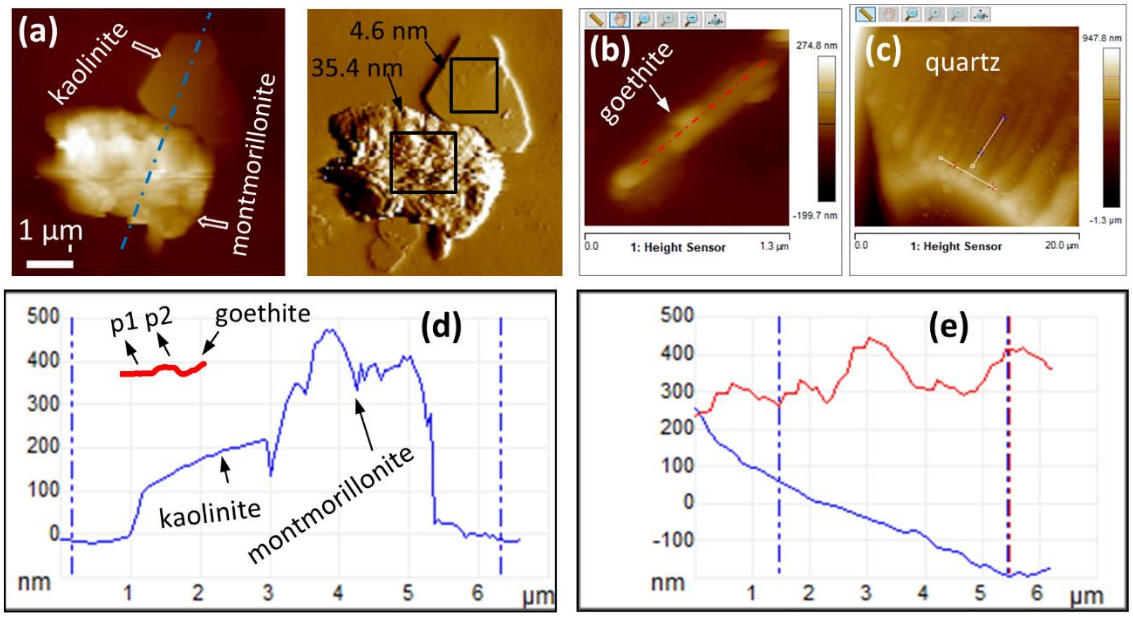

Fig. S-I 2 (a) Height channel (left) and Peak Force Error channel (right) with numbers indicating the respective roughness (R_q) of an AFM map of kaolinite and montmorillonite particles in the highlighted squares chosen to avoid the edges. Height channel of goethite **(b)** and quartz **(c)** particles, **(d)** and **(e)** line scans with position shown in (a), (b) and (c). Montmorillonite is dominated by an irregular morphology which does not purely reflect the lamellar crystal structures as shown in the blue line scan (d). The goethite cluster made of two particles p1, p2 has very smooth appearance (red line scan). The two-line scans across two directions of quartz (e) show high roughness of this mineral.

S-I.2 Creating the tip area-height functions

To check the ability of the proposed inverse imaging method to reflect the real shape of the scanned elements, we applied it to get the topography of standard well-known shapes. In Fig. S-I 3, the measured tip radius of 35 nm of a chemically modified probe inversely scanned by a very sharp characterizer fits to the nominal value of 40 nm. Moreover, the agreement between the shape of the indenter scanned by inverse imaging (Fig. S-I 3b) and the tip impression of a membrane surface indented by the respective indenter (Fig. S-I 3c) is an evidence that the inverse imaging method has a great ability to track shapes with different geometries. To ensure that the mineral is interacting with the cell surface rather than the glue, we scanned the same cell first by the glue tip and then by the same tip after modification (Fig. S-I 3d and e). It is clear that the cell shape got sharper after tip modification.

In nanoindentation research, the slight deviation between the engineered and ideal self-similar shape of the indenter is corrected by the "tip-area-function" which is obtained by making several indentation experiments with increasing loading force and deducing the contact area from the sample stiffness as a relation to the indentation depth (deformation)[1]. This pioneer work thus established a depth-dependent-indenter-shape calibration on a known sample before using the respective indenter for measuring the unknown sample. This was inspiring for AFM researchers to apply similar approaches to evaluate e.g. not only the contact area but also the different mechanical properties of the different membrane layers forming a bacterial cell[2]. It is still required, however, to use a flat surface for calibration and to approximate the shape of the cell to e.g. sphere or cylinder which do not fit our study of interaction forces between two irregular surfaces at the tip and the sample.

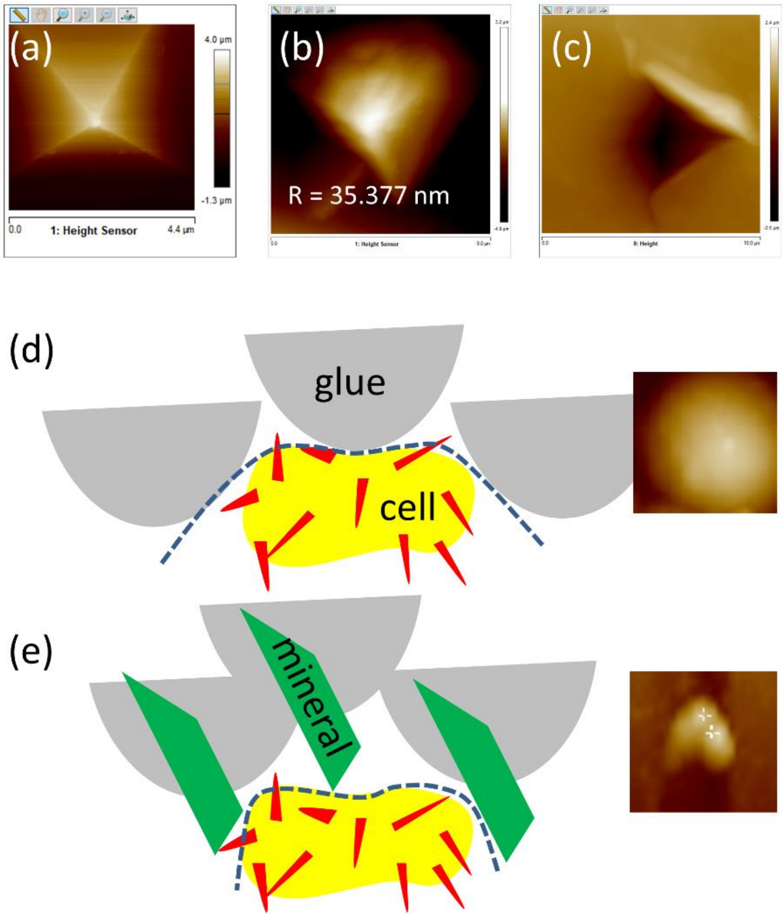

Fig. S-I 3 Inverse image of **(a)** chemically modified probe and **(b)** AFM indenter. **(c)** Image of a membrane surface after indentation experiment with the indenter in (b). AFM image with sketches illustrating interaction between **(d)** a glue tip and a cell and **(e)** the same tip after modification and the same cell.

We thus used inverse imaging to create a 3D image of the modified tip[3]. With height step of 2 nm, the inverse images of the tips were analyzed using the roughness function of the NanoScope Analysis software (version 2.0, Bruker) (Fig. S-I 4a and b). The resultant 3D area of the data was plotted against the deformation D (Fig. S-I 4c, top). As we use another technique to obtain the tip shape, the term "tip area-height function" represents a correlation between the 3D area of the "irregular" modified probe (with no geometrical approximation) and the tip height (deformation). We also plotted the root mean roughness R_q (formula in main article) in a relation to D in order to check if the local irregularities within the mineral tips have various values among the different tips.

We considered the stiffness of cells negligible compared to that of the minerals as assumed elsewhere.[4,5] We already evaluated the mechanical stability of the glue body during AFM interaction which allows us to assume that the glue structure is much harder than the cells[3]. Taking into account the high elastic response of the cell wall, whose mechanical structure can be understood as an inflated balloon[6], we assume that its shape conforms to the local irregularity of the mineral at the cell-mineral interface (here local irregularity refers to crystal defects e.g. the steps shown in Fig. 5f in main article and the section on goethite surface in Fig. S-I 18). In addition, unlike the use of very high loading forces and/or the use of standard sharp AFM tip which lead to overstretching of the cell membrane and thus overestimation of the contact area, the relatively dull mineral tip shapes and low force loads used in this work allow us to assume that the cell membrane is able to stretch enough and cover the full 3D area of the mineral tip which gets in contact with the cell surface[7,8].

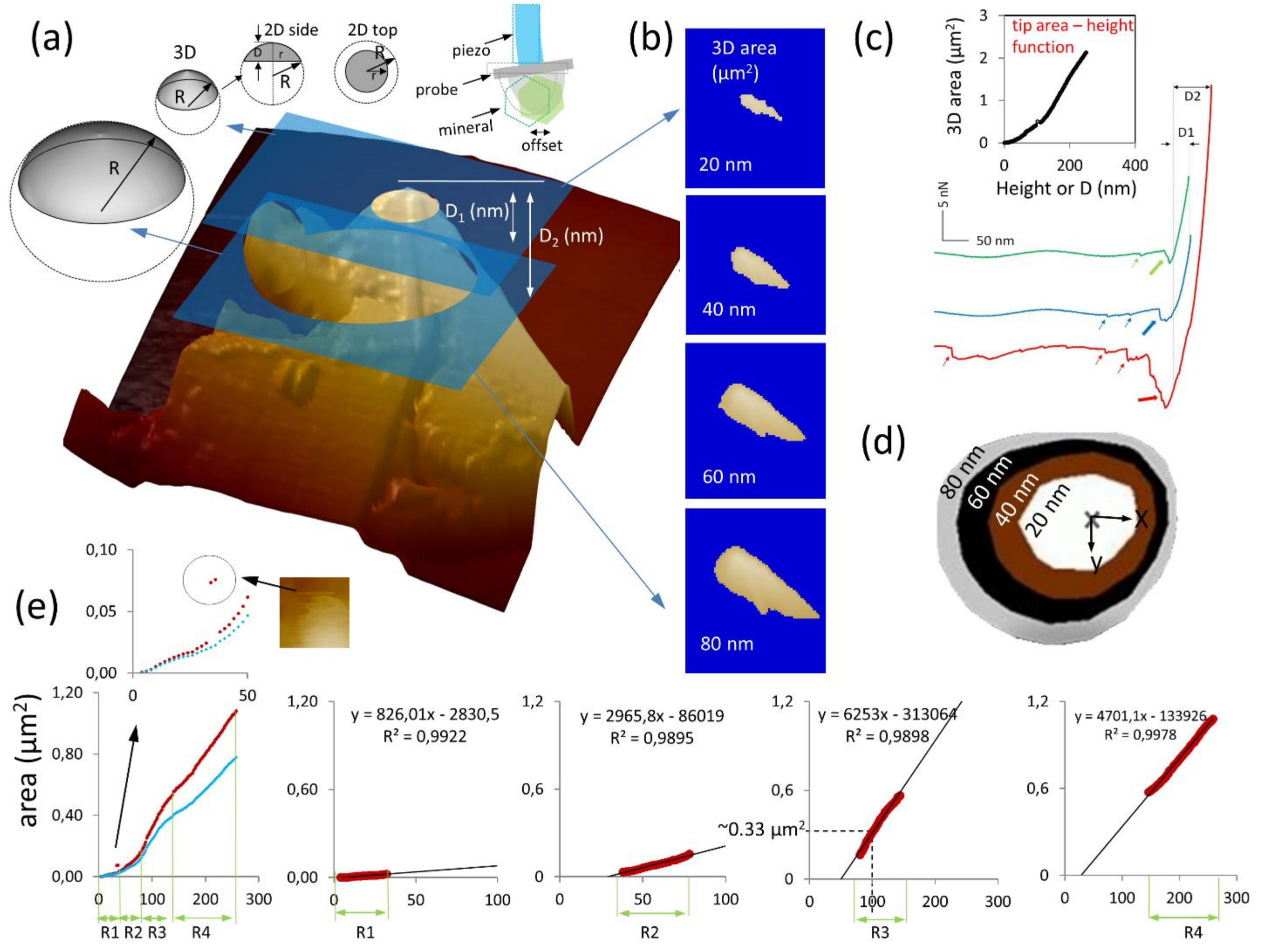

Fig. S-I 4 Our improved technique to infer the contact area of the mineral tips. **(a)** inverse AFM image of the kaolinite modified probe with 3D caps of equivalent 3D area of the mineral tip at the two deformation levels D1 and D2, view of the smaller cap at D1 from top and side orientations and finally an illustration of the shape of the modified probe with little lateral displacement of the piezo, **(b)** 3D area (from the top it appears as 2D area) of the kaolinite particle at increasing height steps from 20 to 80 nm, **(c)** tip area-height function which relates the 3D area and the deformation D (top) and examples of pull-off curves (bottom) of cell-mineral interactions which highlight D1 and D2, **(d)** representative shape of a montmorillonite particle at increasing height steps from 20 to 80 nm, and **(e)** classification of the area-height function into height regimes in which the data show a linear relationship with the dashed line showing an example of how to get the 3D area at D of 100 nm. The outliers (circle) show how the 3D data get affected by line artifacts of the inverse image, which is not the case in the 2D area (blue curve). Such outliers are deleted.

We also neglected the effect of the limited inclination of the AFM probe induced by bending of the piezo with XY offsets on possible changes in the orientation of the terminal part of the probe (inset in Fig. S-I 4a). Thus, the contact area is simply defined as the surface area of the portion of the mineral which gets in contact with the planar cell surface under the loading force. As the tip area-height functions were nonlinear, they were divided into height regimes in which the data show a linear relationship (Fig. S-I 4e). The 3D area is deduced from substituting the deformation value (e.g. D1 or D2 in Fig. S-I 4c) in the tip area-height function (Fig. S-I 4e). The deformation values were manually estimated by placing one marker at the contact point of the approach curve and the second one at the point of maximum force of the retract curve of the Force-Separation curve (FD curve) using the NanoScope Analysis software. The choice of the retract curve for the point of maximum force is important because for retract curves acquired with 1 s holding time, the loading force (force setpoint) was sometimes changed compared to the approach curves possibly due to Z draft of

the piezo and/or creep of the cell surface. An example of how to get the 3D area at deformation of 100 nm is shown in Fig. S-I 4e. In order to reflect the real mineral tip orientation and shapes, we applied no filters to the inverse images. However, it was important to correct for the little line or point scan artifacts (clear increase or reduction of height data at single lines or points of the inverse images). Thus, the projected area (2D area) which is unaffected by these artifacts was used as reference to exclude the outlier points of the 3D area as shown by the inset in (Fig. S-I 4e). By comparing our estimation of the contact area with the commonly used Hertz's theory[8,9], we directly get the 3D area of the interacting geometry without the need to assume a spherical tip shape.

To check the reliability of the tip sizes estimated by inverse imaging by a comparison with the size of interaction from other works and to see if the XDLVO theory which requires approximation of the tip shape to a sphere can predict the same trends as detected by AFM, the irregular shapes of the mineral tips were approximated to spherical caps that have the same 3D area as the mineral tip (Fig. S-I 4a).

S-I.3 3D area of CMI

The contact radii of the 5 mineral tips, calculated based on the average deformation of the biological surfaces, being 1517, 217, 327, 228 and 553 nm for the kaolinite tips, 304, 420, 209, 224 and 120 nm for the montmorillonite tips and 60, 244, 185, 55 and 165 nm for the goethite tips generally agree with the range of hydrodynamic radii calculated in another work[10] with 652 ± 55 nm, 302 ± 1 nm (or 250 nm[11]) and 207 ± 4 nm, respectively. The radii of the quartz tips of 1015, 2165, 413, 134 and 693 nm vary in a wider range and are on average larger than the radii of the clay mineral tips owing to the larger particle size and more irregular particle shape of quartz.

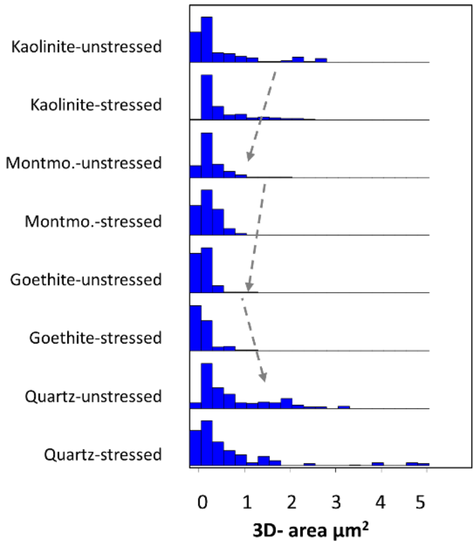

Fig. S-I 5 Distribution of the 3D areas with the arrows showing the trend of increase or decrease between the unstressed cells (which is the same for stressed cells) and the various minerals.

S-I.4 Deconvolution of irregular shaped particles

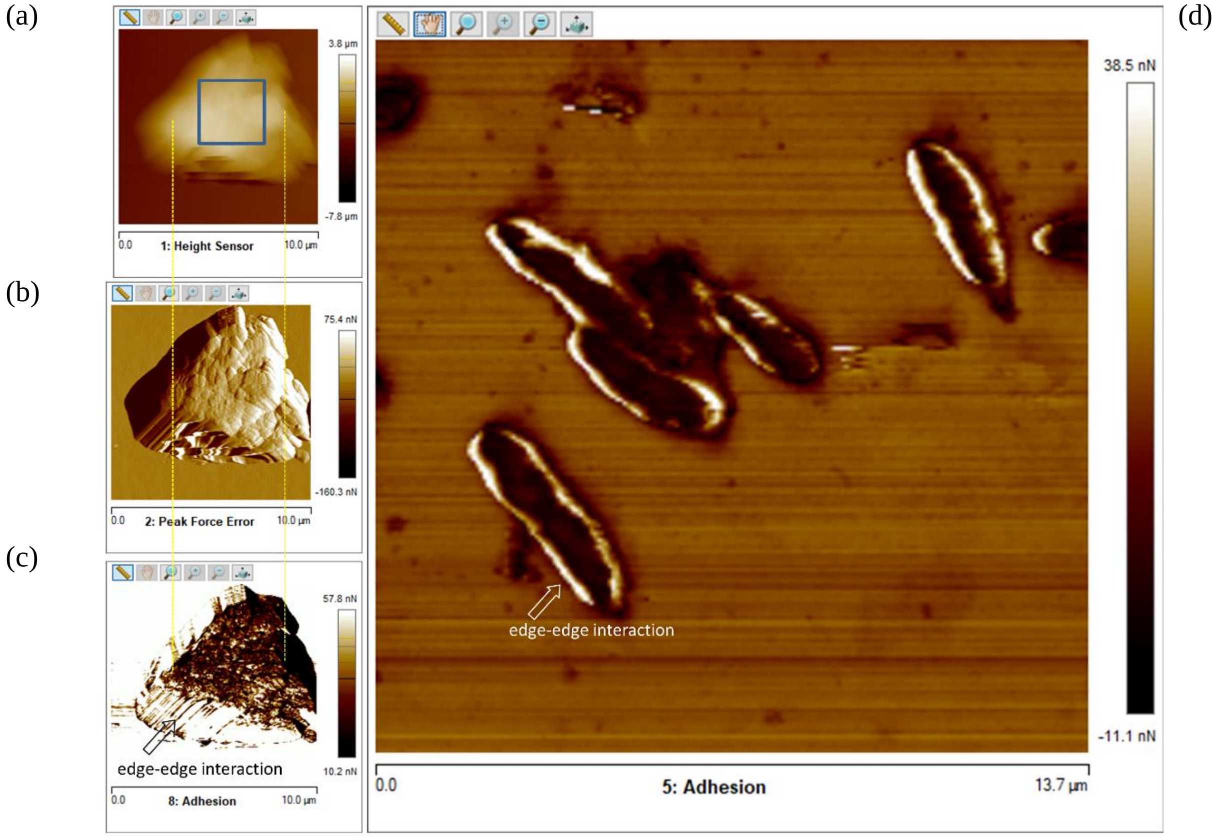

Fig. S-I 6 (a) AFM height, **(b)** PeakForce Error, and **(c)** adhesion map of a montmorillonite particle obtained by a sharp tip showing the particle surface between the two dashed lines and the edge-edge interaction outside. Blue frame shows the area considered for R_q estimation excluding side areas. **(d)** Adhesion map of some cells measured by sharp tip (right). In is evident that the adhesion force gets higher at the edge of the particle and cells which might be due to the enhanced contact area between the side of the probe and the side of the mineral or cell. Such “side effects” were avoided in this work by making FD curves on the cell centers.

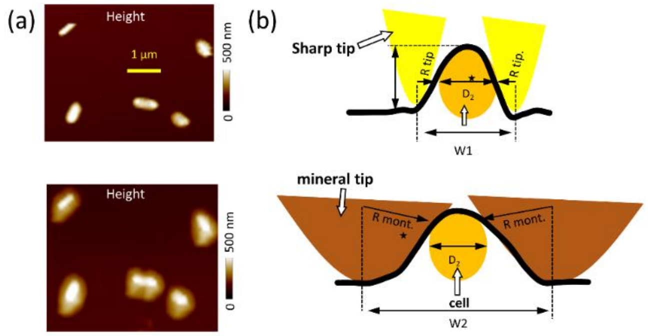

Fig. S-I 7 (a) AFM height map of cells made by a sharp tip (top) and a montmorillonite tip (bottom) and **(b**) sketches showing how the bacterial size gets enlarged because of tip-sample deconvolution.

S-I.5 Cell-mineral interaction

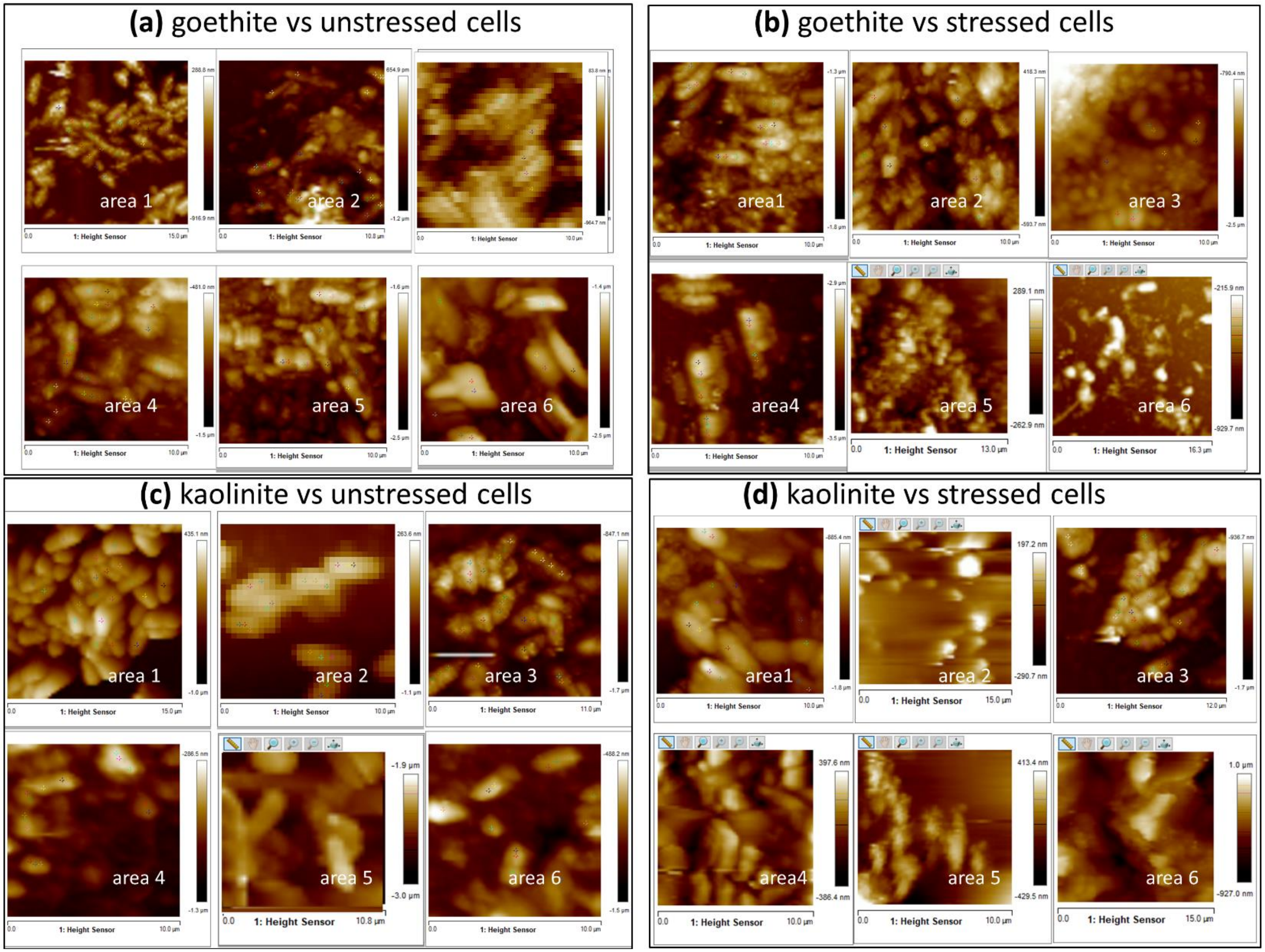

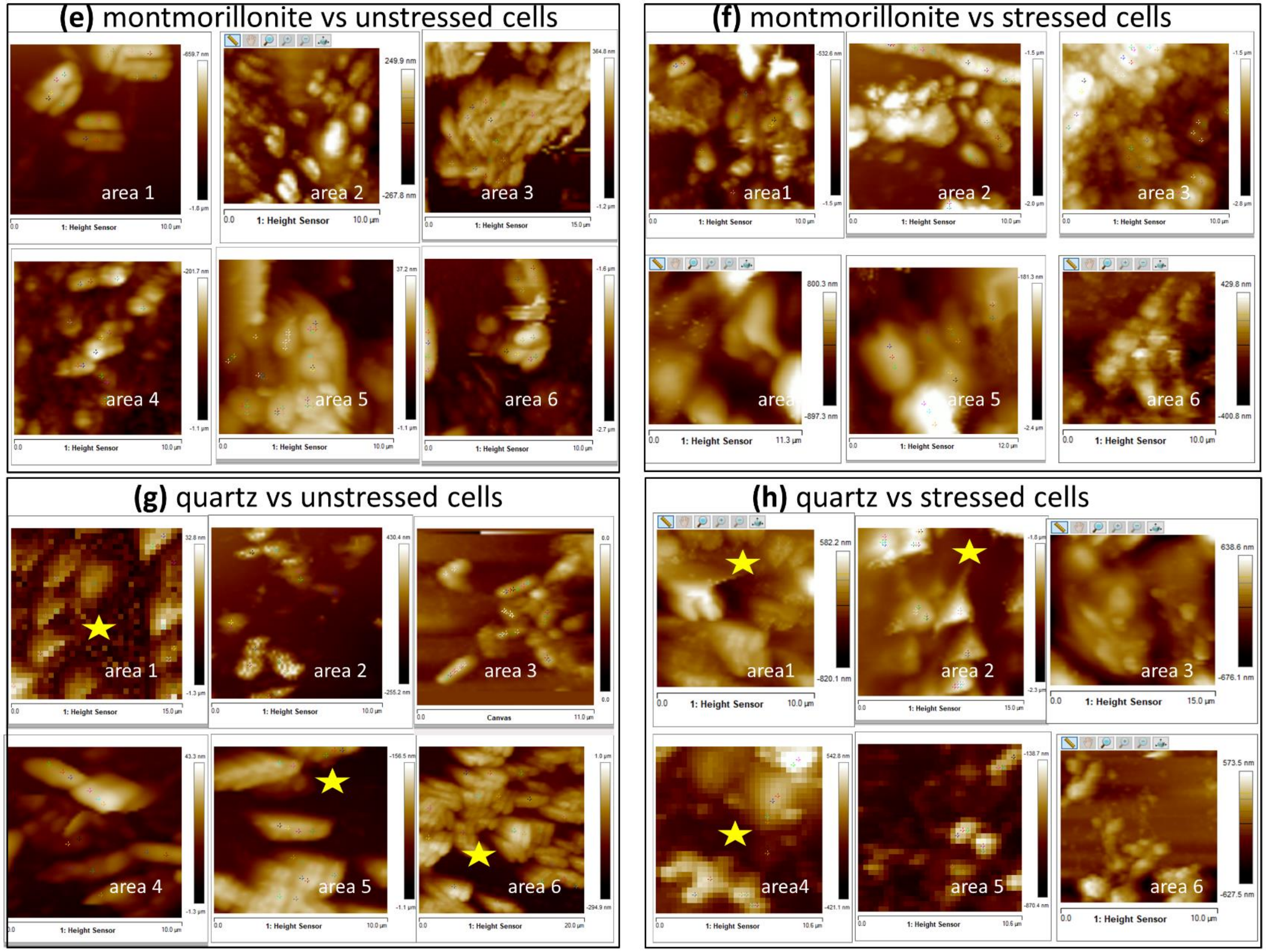

Fig. S-I 8 Examples of interactions between **(a, c, e, g)** unstressed and **(b, d, f, h)** stressed bacterial cells and probes modified with **(a, b)** goethite, **(c, d)** kaolinite, **(e, f)** montmorillonite and **(g, h)** quartz with marks indicating the positions of FD curves acquisition. All images were made in 10 mM KNO_3 solution. For some cases (stars on the images), the complex shape of the quartz tips produced repetitive cell structure.

S-I.6 Adhesion force, deformation and surface area versus loading force

In most cases, the adhesion forces increase linearly with increasing loading force applied by the mineral tips on the studied cell surfaces. In line with an increasing deformation and thus increasing contact area with increasing loading force, the adhesion forces also increase linearly with increasing contact area (Fig. S-I 9).

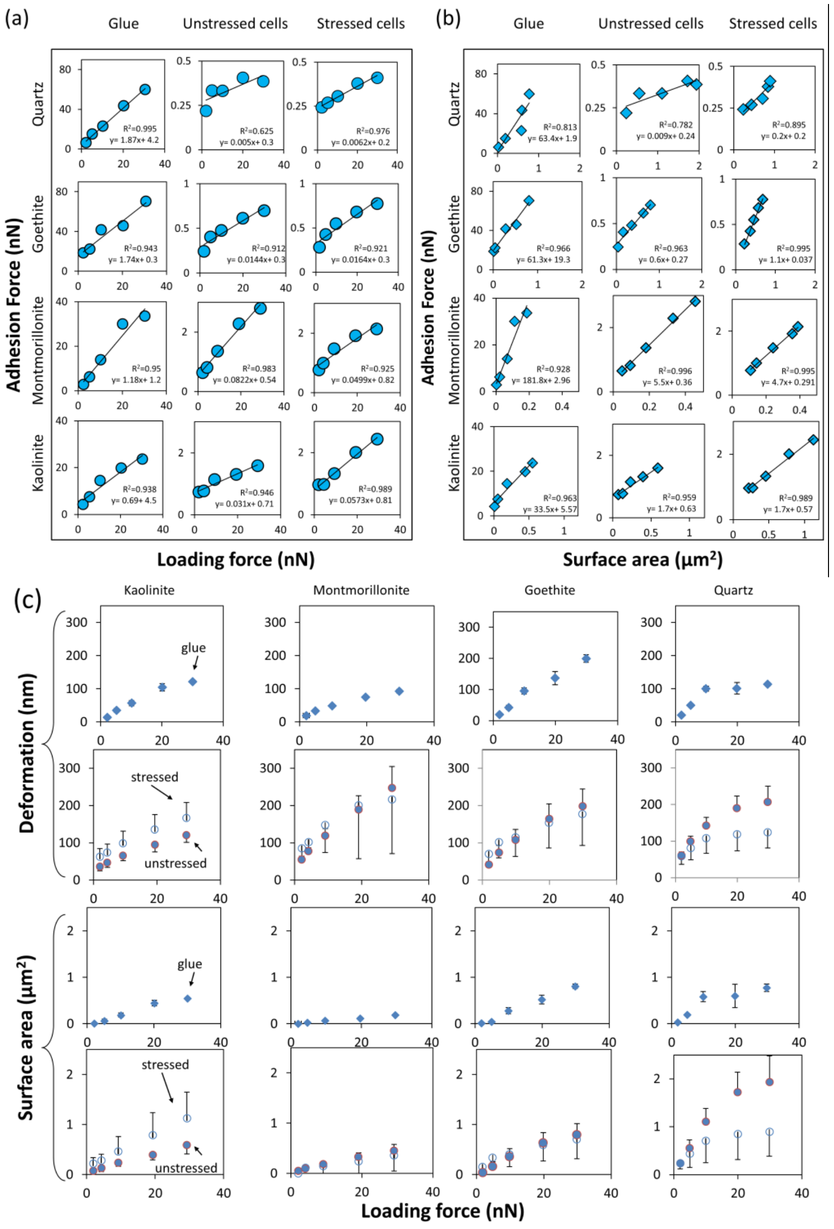

Fig. S-I 9 Linear fitting of the relationship between mean adhesion force and **(a)** loading force and **(b)** calculated contact area for the interaction between cantilevers modified with four different mineral particles (each in one row) against reference glue surfaces, unstressed and stressed cell surfaces (each in one column) and **(c)** dependence of deformation (top) and surface area (bottom) on the loading force. Data are given as arithmetic means of 10 curves performed on the centers of several cells (2 outliers of quartz were removed). If applied, error bars represent the standard deviations in one direction. Measurements were made in 10 mM KNO_3 solution. The contact time with the cell surfaces was 1 s and with the glue surfaces was 0 s.

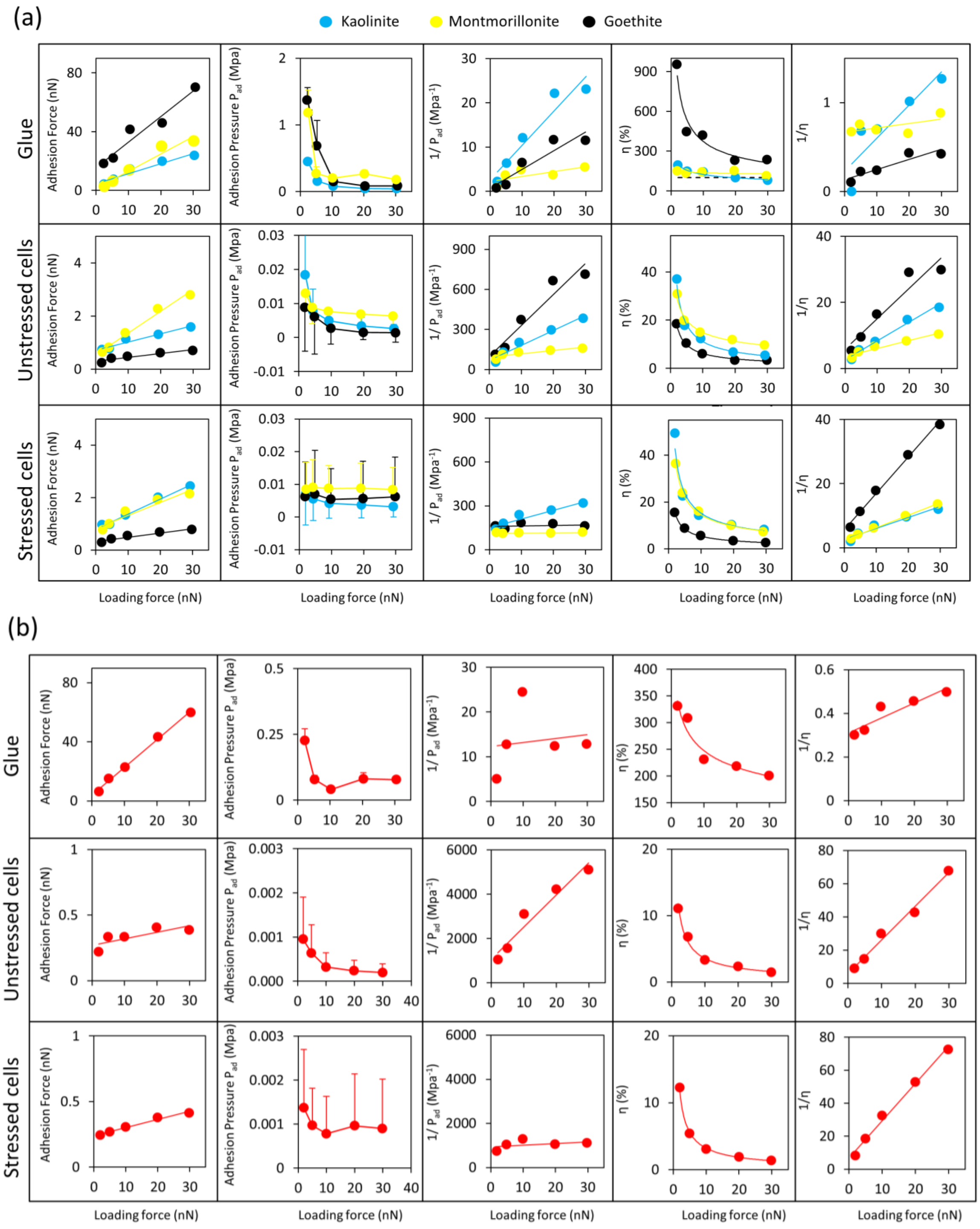

Fig. S-I 10 Dependence of adhesion force, adhesion pressure, inverse mean of adhesion pressure, adhesion efficiency η (%), and inverse mean of adhesion efficiency on the loading force for interactions of **(a)** the small sized minerals kaolinite, montmorillonite and goethite and **(b)** quartz versus the stressed and unstressed cells and glue surfaces. Note the dashed line representing 100% efficiency at which the adhesion force equals the loading force. Data are given as arithmetic means of 10 curves performed on the centers of a couple of cells (2 outliers of quartz were removed). The error bars represent the standard deviations in positive direction.

The positive correlation of the adhesion forces with the 3D contact areas of the four minerals used in this work (Fig. S-I 9b) is attributed to a stronger binding capacity with increasing number of contact points between the tip and the cell surface[12]. The deformation, and thus also the produced surface area, sometimes has different levels or deviations for the stressed and unstressed cells which might be due to differences in the cell elasticity for these two bacterial communities. Comparing interaction between kaolinite and the cells with quartz and the cells, the effect of stress on deformation is not reproducible. This emphasizes the urgent need to correct adhesion forces by the respective contact area to facilitate direct comparison of adhesion pressures of minerals toward stressed and unstressed cells.

Besides having lower standard deviation, the resultant adhesion forces towards the glue surface are almost 1-2 orders of magnitude larger than those towards the cell surfaces. The positive correlation of the adhesion forces with the 3D contact areas of the four minerals used in this work (Fig. S-I 9b) is attributed to a stronger binding capacity with increasing number of contact points between the tip and the cell surface[12]. The deformation, and thus also the produced surface area, sometimes has different levels or deviations for the stressed and unstressed cells which might be due to differences in the cell elasticity for these two bacterial communities. Comparing interaction between kaolinite and the cells with quartz and the cells, the effect of stress on deformation is not reproducible. This emphasizes the urgent need to correct adhesion forces by the respective contact area to facilitate direct comparison of adhesion pressures of minerals toward stressed and unstressed cells. For both, cells and glue surfaces, however, the adhesion pressure P_{ad} as well as the adhesion efficiency η non-linearly decay with increasing loading force while their reciprocal values ($1/P_{ad}$; $1/\eta$) increase linearly with the loading force (Fig. S-I 10). However, for the stressed cells $1/P_{ad}$ appeared rather constant with increasing loading force for the interaction with montmorillonite, goethite and quartz.

S-I.7Effect of contact time

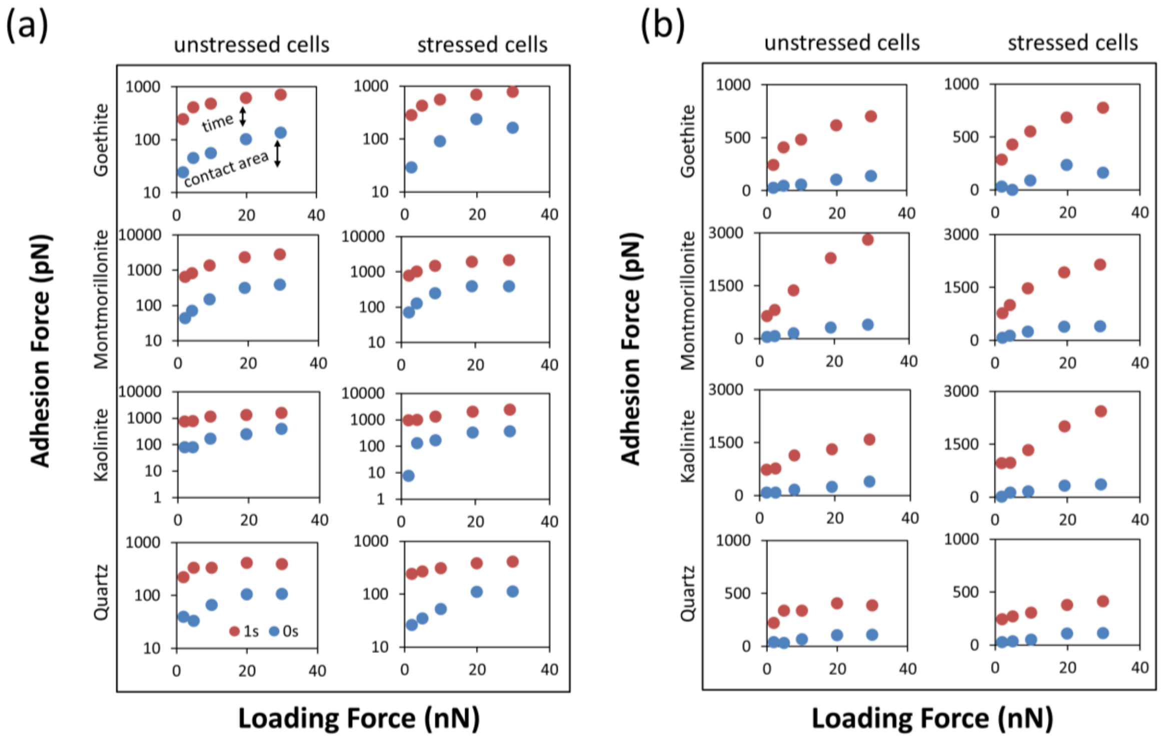

Fig. S-I 11 Dependence of adhesion forces on the loading forces for interactions between mineral particles against unstressed and stressed cells for 0 (blue) and 1 s (red) contact time **(a)** in log scale and **(b)** in normal scale given as arithmetic means of 10 curves performed on the centers of a couple of cells (2 outliers of quartz were removed).

The surface contact time has a significant effect on adhesion strength. Not only the average adhesion forces weakened by almost an order of magnitude as the contact time got reduced from 1 to 0 s (Fig. S-I 11), but also the frequency of adhesion peaks was with 94% - 100% (Fig. S-I 12) and rupture events with 48% - 74% (Fig. S-I 13) considerably higher at 1 s than at 0 s contact time (the frequency of events is calculated as the percentage % of curves that show at least one event from the overall curves).

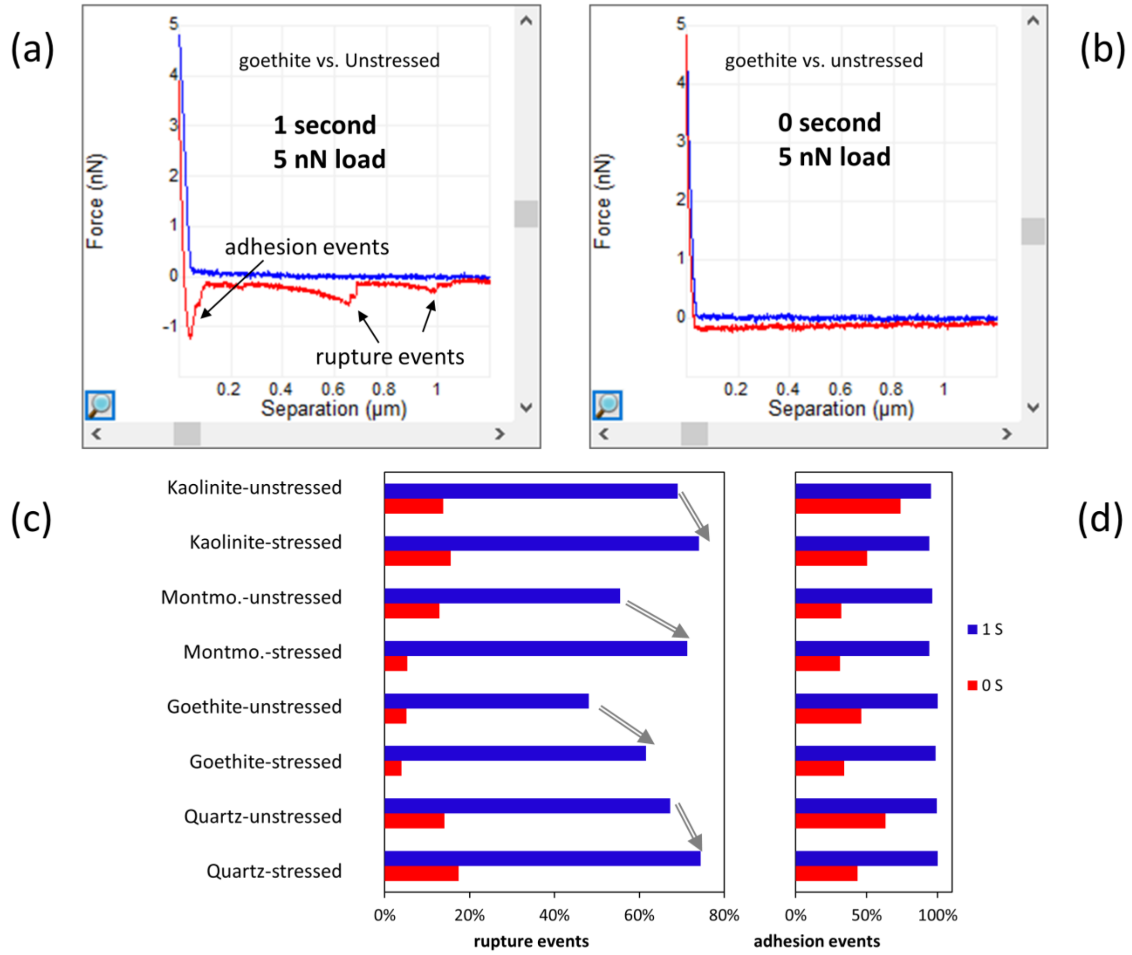

Fig. S-I 12 Examples of FD curves made by goethite probes vs. unstressed cells **(a)** at 1 s and then **(b)** with 0 s contact time. Percentages of CMIs with **(c)** rupture and **(d)** adhesion events of the various cell-mineral pairs. The binding capacity seems to be larger upon stress (double arrows). It is clear that reducing the contact time leads to a reduction in the likelihood of rupture and adhesion events. At 1 s contact time, the high frequency of 94-100% appearance of adhesion peaks in the FD curves together with 48-74% appearance of rupture events shows that the mineral tip indeed interacted with surface biopolymers of the cells.

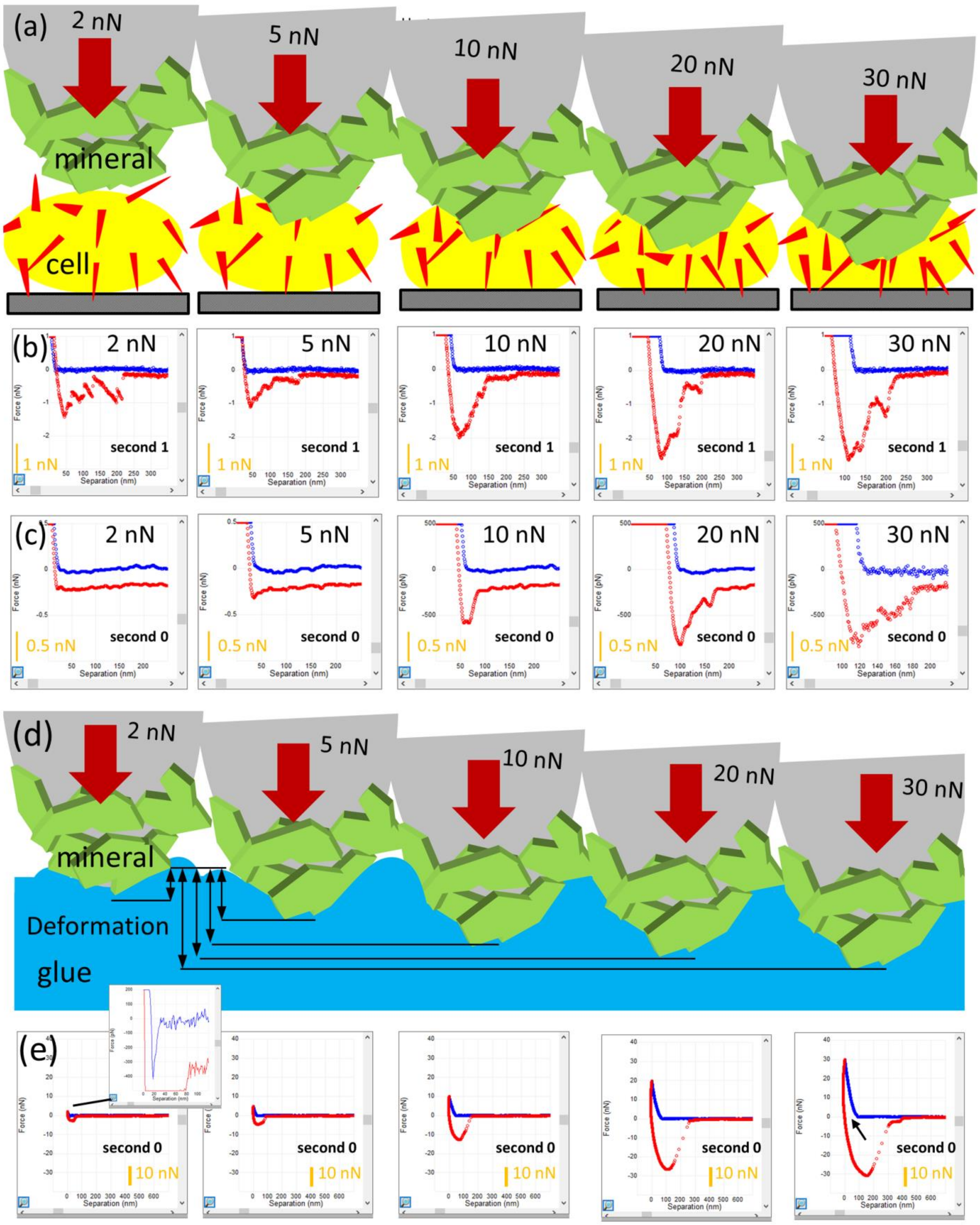

Fig. S-I 13 (a) Sketch of the modified probe interaction with the cell surface at increasing loading force from 2 to 30 nN, FD curves made by kaolinite probe vs. unstressed cells **(b)** at 1 s and then **(c)** with 0 s contact time, **(d)** sketch of the modified probe interaction with glue at increasing loading force from 2 to 30 nN and **(e)** FD curves made by kaolinite probe vs. glue at 0 s contact time. The inset shows jump-to-contact towards the glue.

S-I.8 Box plots of P_{ad}

The high scatter of the P_{ad} among the various probes for the specific cell-mineral interaction (Fig. S-I 14) was expected as a consequence of the different interaction geometries and the different properties of the finite parts of the minerals that get in contact with the cell surface. Therefore, it is important to employ multiple probes to get a reliable data on adhesion pressures of different mineral species and compare them. Using a single probe might reflect the effect of one specific tip geometry and hide the material dependent trend of the overall adhesion behavior detectable only by applying several probes.

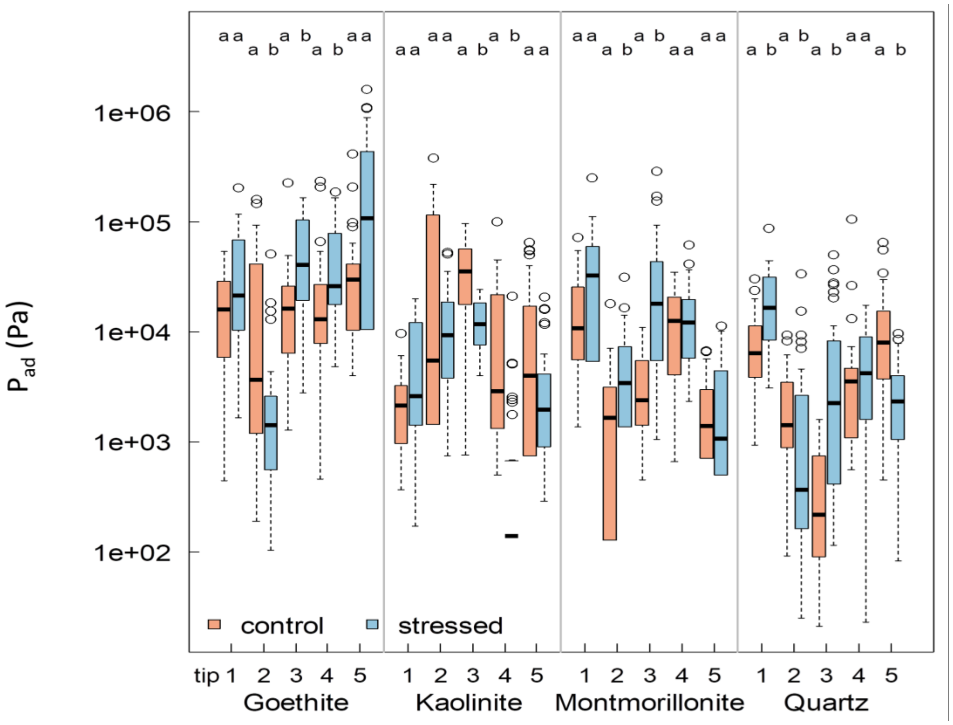

Fig. S-I 14 Box plots of adhesion pressures of cell-mineral interactions between cells of two independent unstressed and three independent stressed *P. fluorescens* cultures and five individual modified probes for each mineral (Fig. 2d in main article) in 10 mM KNO_3 solution. A set of ~30 FD curves for each probe with 1 s holding time and 5 nN applied force was made.

S-I.9 Surface chemical composition

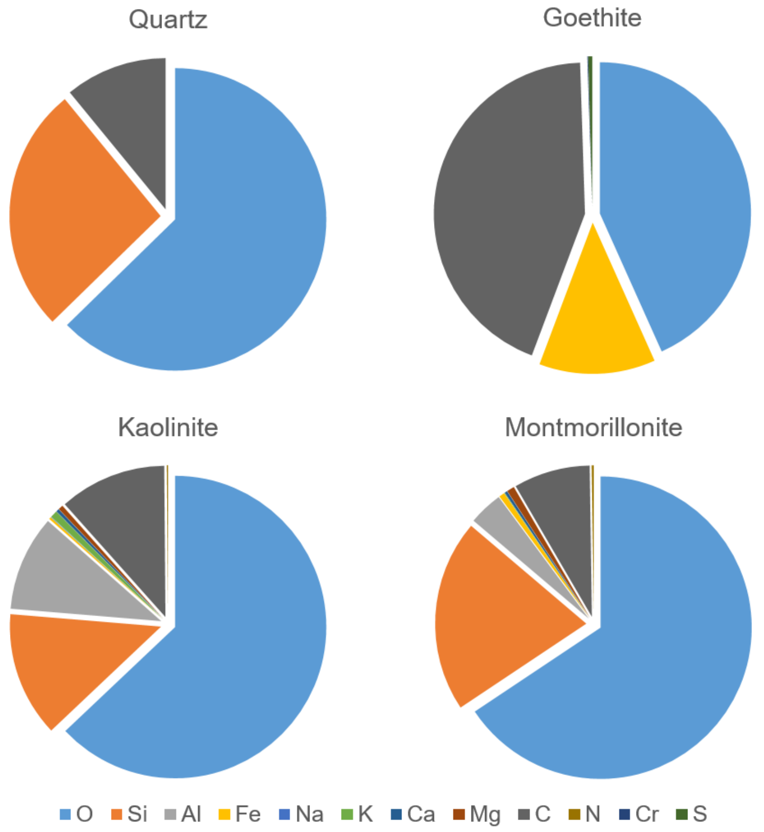

Fig. S-I 15 Surface chemical composition of the minerals. Given are mean values of *n* = 3 measurements.

S-I.10 Cell-mineral interaction energy profiles

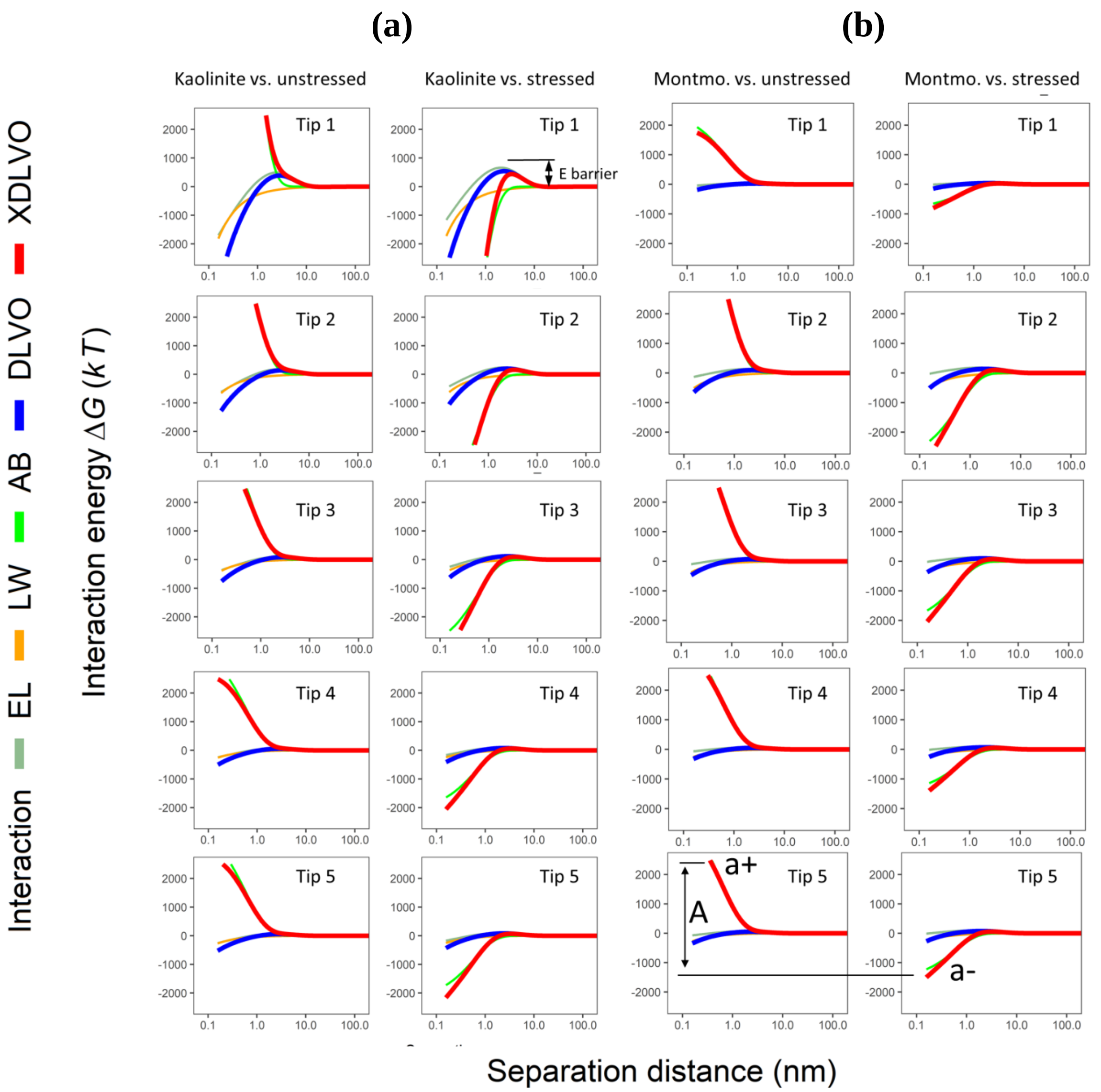

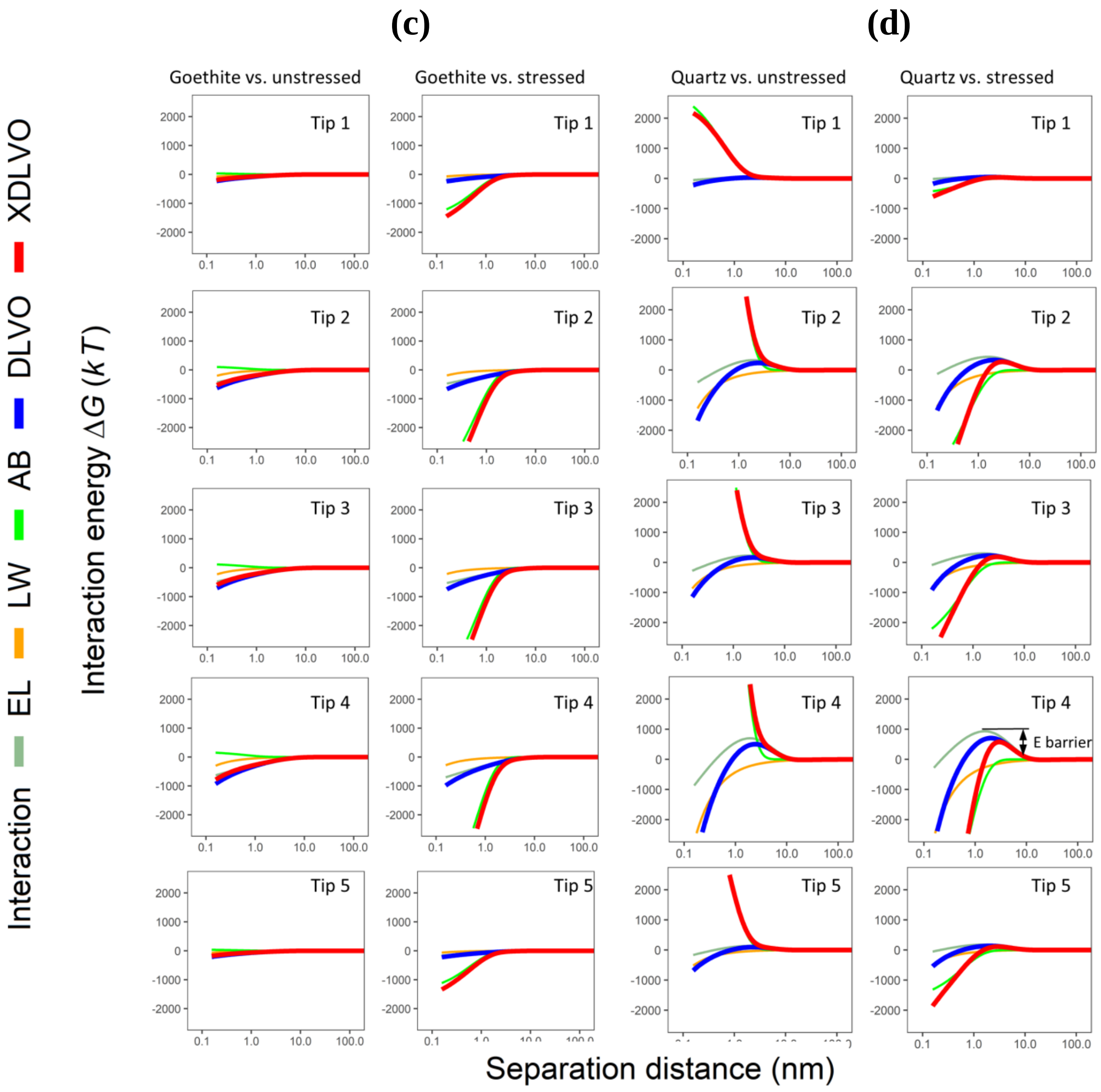

Fig. S-I 16 Energy profiles showing the total interaction energy and its individual components (EL: electrostatic, LW: Lifshitz-van der Waals, AB: acid-base) as a function of separation distance between tips modified with **(a)** kaolinite, **(b)** montmorillonite, **(c)** goethite, and **(d)** quartz and unstressed or stressed bacterial cells in 10 mM KNO_3. Total interaction energy was calculated as sum of EL, LW and AB components (XDLVO) and as sum of EL and LW components, neglecting the AB component (original DLVO).

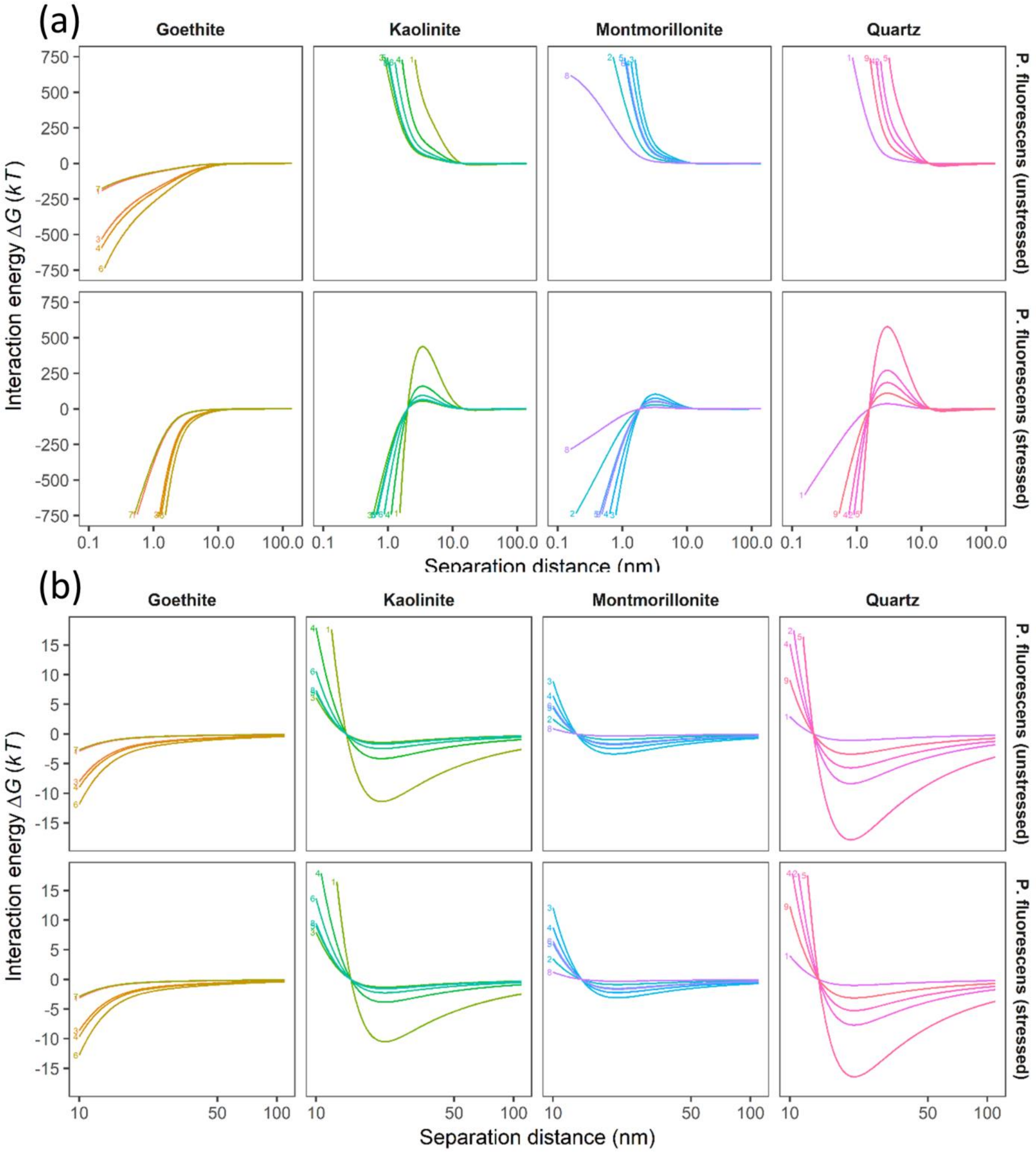

Fig. S-I 17 (a) Energy profiles showing the total interaction energy (XDLVO) as a function of separation distance between the different mineral modified tips and unstressed or stressed bacterial cells in 10 mM KNO_3, **(b)** same as (a) depicting the secondary minima.

S-I.11 Tilt of the mineral particles

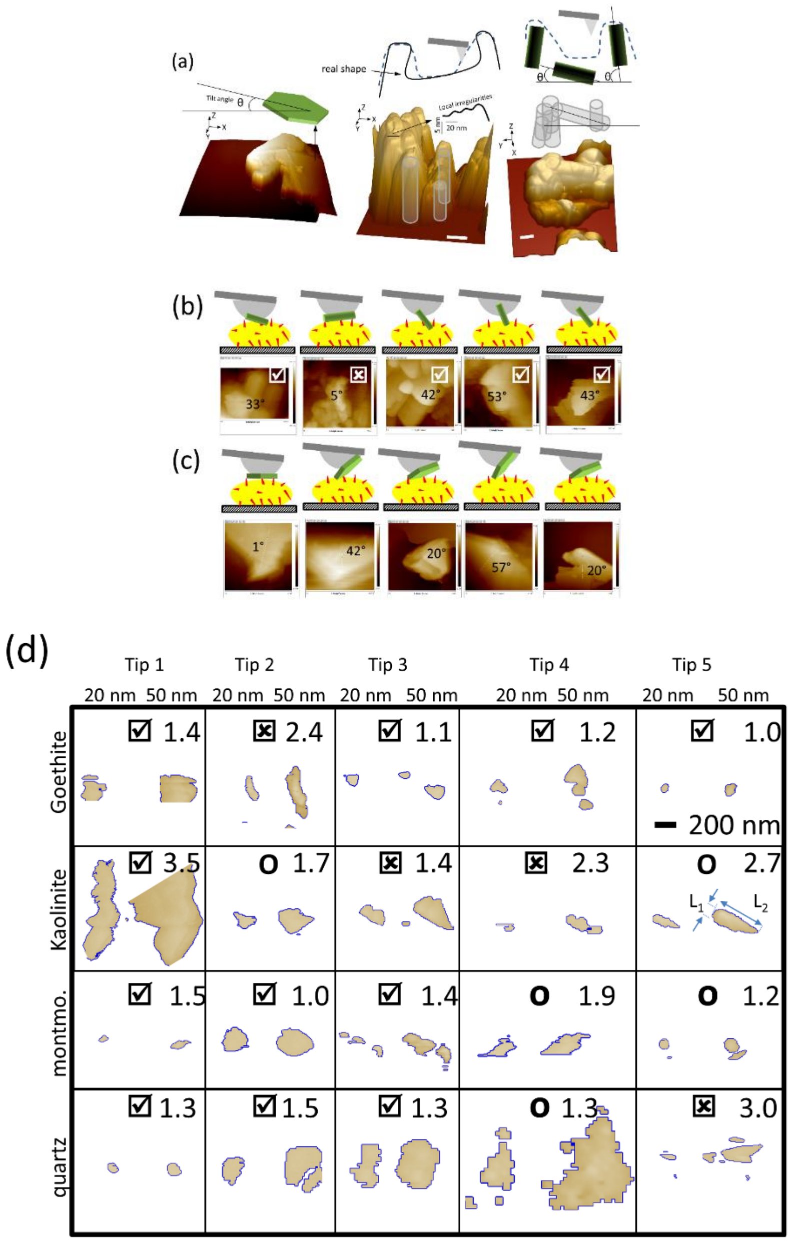

Fig. S-I 18 Estimation of the orientation of the kaolinite and goethite particles fixed at the AFM probes **(a)** AFM inverse images of mineral clusters with reconstruction of the particle vectors **(b)** AFM images of the goethite tips with the tilt angle written beside the particles. The sketches on the top show how the respective probes look like. **(c)** Same as (b) for kaolinite tips and **(d)** symmetry evaluation of the 2D projected shapes of the tips at 20 nm and 50 nm D values. The number in each square represents the aspect ratio (L_2/L_1) of the respective tip at D = 20 nm which is qualitatively the same for D = 50 nm. In the case of discontinuous areas for the same tip, aspect ratio is ($\sum L_2/\sum L_1$). Though we work in simplified 2D space, the last expression was used because the interacting force of single tip toward a flat surface equals the force produced by several tips with same total volume[13]. ☑ and ☒ indicate agreement and contradiction of the response to stress studied by AFM and XDLVO (main article), respectively. (O) indicates no match.

S-I.12 Unbinding force

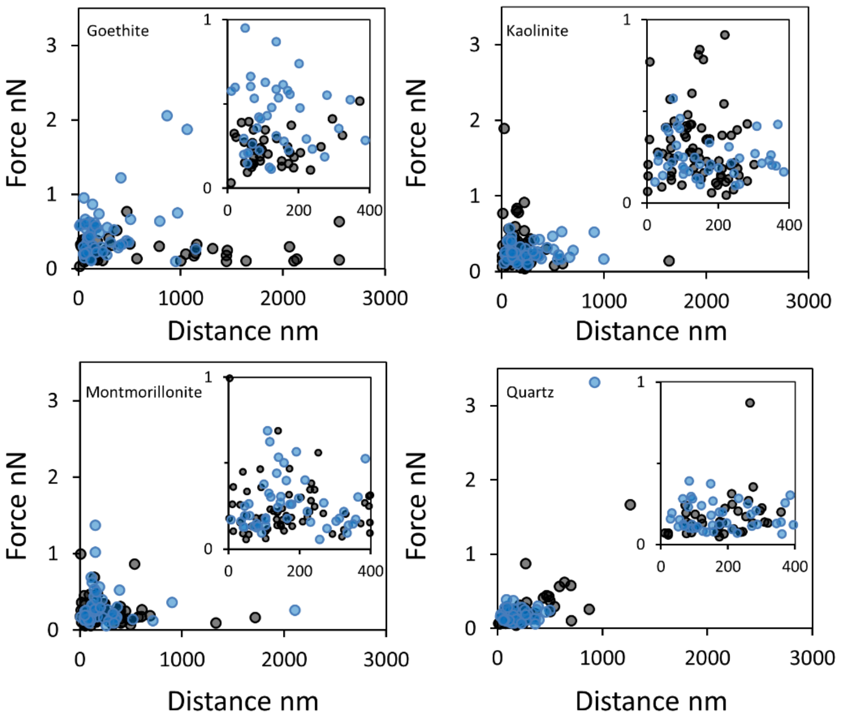

Fig. S-I 19 Force of the last rupture (unbinding) as a function of the distance from the sample surface for interaction between each of the minerals versus unstressed (black) or stressed (blue) cells. The insets show data at narrow range.

S-I.13 Relocation system

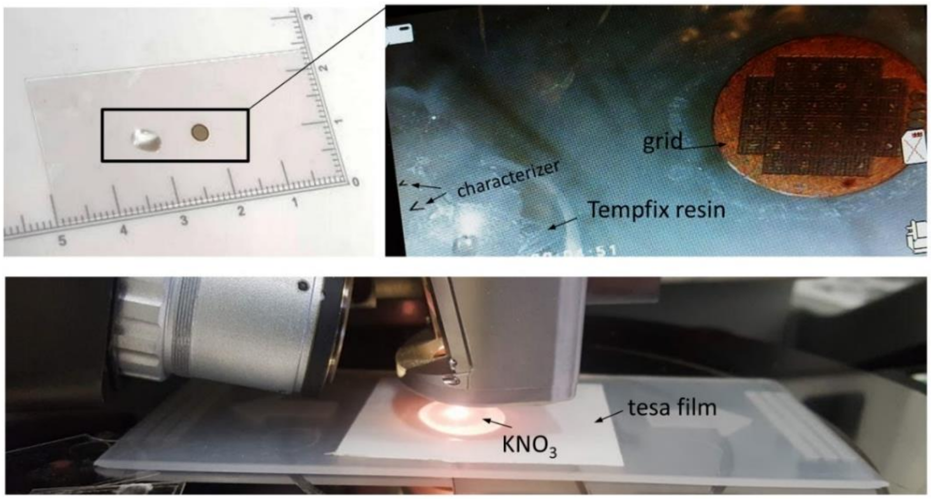

Fig. S-I 20 Camera image with the length units in cm (top left) with the zoom (top right) of the relocation system. The characterizers are fixed on the glue spot as shown in https://www.youtube.com/watch?v=0KIK7pqdpLY. The finder grid is fixed on the backside of the cover glass in order to avoid its interaction with the AFM tip. The cells are attached on the top of the grid (frontside) but cannot be seen at this magnification. Image (bottom) of the tesa film used as reference surface.

S-I.14 Functionalization of tipless cantilevers with glue

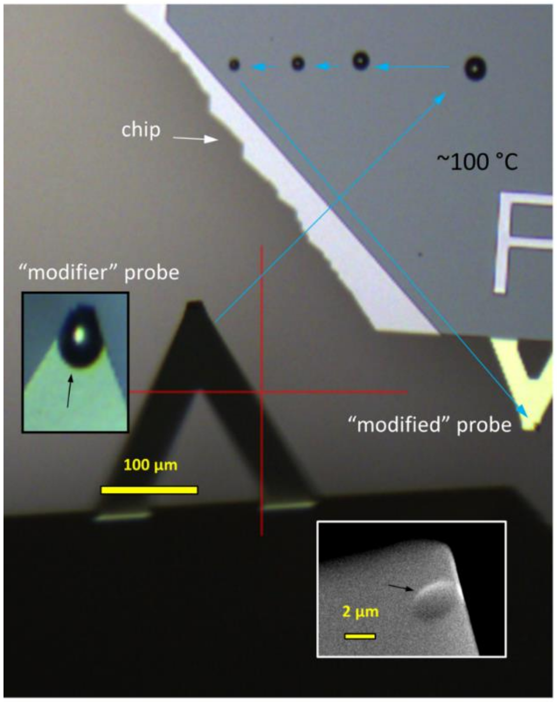

Fig. S-I 21 Light image of the "modifier" probe attached to AFM with the inset (left) showing its frontside and the chip of the "modified" probe fixed at the heated AFM stage. As the "modifier" probe contacted the chip at several points (blue arrows), the glue size gets smaller and smaller. By this procedure the size of the glue spot at the end of the tipless cantilevers was fitted to the size of the clay minerals as shown by the SEM image in the inset (bottom right).

S-I.15 Results from statistical tests

degree of freedom	p value Shapiro-Test control	p value Shapiro-Test stressed	p value Levene-Test	mean value pN µm^{-2} control	mean value pN µm^{-2} stressed	p value Wilcoxon-Rank -Sum-Test
**Goethite**						
305	2.6429E-20	1.1878E-21	0.00046967	30279.6859	94021.4901	0.04061654
**Kaolinite**						
304	6.0836E-20	5.0732E-14	2.2988E-05	24548.6579	7666.01948	0.01096007
**Montmorillonite**						
303	3.808E-17	4.5635E-20	9.087E-05	7395	21078.6933	0.00024083
**Quartz**						
303	3.2612E-21	6.6089E-17	0.15334386	6300.25	8297.26174	0.20097996

degree of freedom	p value Shapiro-Test Mineral 1	p value Shapiro-Test Mineral 2	p value Levene-Test	mean value pN µm^{-2} Mineral 1	mean value pN µm^{-2} Mineral 2	p value Wilcoxon-Rank -Sum-Test
**Goethite - Kaolinite - Control**						
306	2.6429E-20	6.0836E-20	0.80416527	30279.6859	24548.6579	7.7942E-05
**Goethite - Kaolinite - Stress**						
303	1.1878E-21	5.0732E-14	5.8263E-06	94021.4901	7666.01948	4.4384E-15
**Goethite - Montmorillonite - Control**						
309	2.6429E-20	3.808E-17	3.3354E-06	30279.6859	7395	4.3819E-15
**Goethite - Montmorillonite - Stress**						
299	1.1878E-21	4.5635E-20	0.00016703	94021.4901	21078.6933	1.3124E-06
**Goethite - Quartz - Control**						
310	2.6429E-20	3.2612E-21	1.6612E-06	30279.6859	6300.25	2.1987E-21
**Goethite - Quartz - Stress**						
298	1.1878E-21	6.6089E-17	9.9062E-06	94021.4901	8297.26174	7.2205E-15
**Kaolinite - Montmorillonite - Control**						
305	6.0836E-20	3.808E-17	1.4564E-05	24548.6579	7395	0.01292108
**Kaolinite - Montmorillonite - Stress**						
302	5.0732E-14	4.5635E-20	0.00014587	7666.01948	21078.6933	0.00105994
**Kaolinite - Quartz - Control**						
306	6.0836E-20	3.2612E-21	7.5038E-06	24548.6579	6300.25	0.00019206
**Kaolinite - Quartz - Stress**						
301	5.0732E-14	6.6089E-17	0.52608528	7666.01948	8297.26174	0.72717217
**Montmorillonite - Quartz - Control**						
309	3.808E-17	3.2612E-21	0.56808103	7395	6300.25	0.09747187
**Montmorillonite - Quartz - Stress**						
297	4.5635E-20	6.6089E-17	0.00054964	21078.6933	8297.26174	0.00040236

S-I.16 References

1. Oliver, W. C. & Pharr, G. M. An improved technique for determining hardness and elastic modulus using load and displacement sensing indentation experiments. *Journal of Materials Research* **7**, 1564–1583 (1992).
2. Chen, Y., Norde, W., van der Mei, H. C. & Busscher, H. J. Bacterial cell surface deformation under external loading. *mBio* **3**, e00378-12 (2012).
3. Abu Quba, A. A., Schaumann, G. E., Karagulyan, M. & Diehl, D. Quality control of direct cell–mineral adhesion measurements in air and liquid using inverse AFM imaging. *RSC Advances* 5384–5392 (2021) doi:10.1039/d1ra00110h.
4. Perry, C. C., Weatherly, M., Beale, T. & Randriamahefa, A. Atomic force microscopy study of the antimicrobial activity of aqueous garlic versus ampicillin against Escherichia coli and Staphylococcus aureus. *Journal of the Science of Food and Agriculture* **89**, 958–964 (2009).
5. Zhang, W., Stack, A. G. & Chen, Y. Interaction force measurement between E. coli cells and nanoparticles immobilized surfaces by using AFM. *Colloids Surf B Biointerfaces* **82**, 316–324 (2011).
6. Lan, G., Wolgemuth, C. W. & Sun, S. X. Z-ring force and cell shape during division in rod-like bacteria. *Proc. Natl. Acad. Sci. U.S.A.* **104**, 16110–16115 (2007).
7. Lucel, S. Atomic Force Microscopy indentation of living cells. *Microscopy: Science, Technology* 433–440 (2011).
8. Sokolov, I., Dokukin, M. E. & Guz, N. V. Method for quantitative measurements of the elastic modulus of biological cells in AFM indentation experiments. *Methods* **60**, 202–213 (2013).
9. Lau, P. C. Y., Dutcher, J. R., Beveridge, T. J. & Lam, J. S. Absolute Quantitation of Bacterial Biofilm Adhesion and Viscoelasticity by Microbead Force Spectroscopy. *Biophysical Journal* **96**, 2935–2948 (2009).
10. Huang, Q., Wu, H., Cai, P., Fein, J. B. & Chen, W. Atomic force microscopy measurements of bacterial adhesion and biofilm formation onto clay-sized particles. *Scientific reports* **5**, 16857 (2015).

11. Feng, B. *et al.* AFM measurements of Hofmeister effects on clay mineral particle interaction forces. *Applied Clay Science* **186**, 105443 (2020).
12. Xu, L.-C. & Logan, B. E. Adhesion forces between functionalized latex microspheres and protein-coated surfaces evaluated using colloid probe atomic force microscopy. *Colloids Surf B Biointerfaces* **48**, 84–94 (2006).
13. Walz, J. Y. The effect of surface heterogeneities on colloidal forces. *Adv. Colloid Interface Sci.* **74**, 119–168 (1998).
